# Nuclear IL-33 restrains the early conversion of fibroblasts to an extracellular matrix-secreting phenotype

**DOI:** 10.1038/s41598-020-80509-5

**Published:** 2021-01-08

**Authors:** Francesca Gatti, Sobuj Mia, Clara Hammarström, Nadine Frerker, Bjarte Fosby, Junbai Wang, Wojciech Pietka, Olav Sundnes, Johanna Hol, Monika Kasprzycka, Guttorm Haraldsen

**Affiliations:** 1 Department of Pathology, University of Oslo and Oslo University Hospital, PO Box 4950, 0424 Rikshospitalet, Norway; 2 K.G. Jebsen Inflammation Research Centre, University of Oslo and Oslo University Hospital, Rikshospitalet, Norway; 3 Department of Surgery, University of Oslo and Oslo University Hospital, Rikshospitalet, Norway

**Keywords:** Renal fibrosis, Interleukins

## Abstract

Interleukin (IL)-33 is a cytokine that appears to mediate fibrosis by signaling via its receptor ST2 (IL-33R/IL1RL1). It is also, however, a protein that after synthesis is sorted to the cell nucleus, where it appears to affect chromatin folding. Here we describe a novel role for nuclear IL-33 in regulating the fibroblast phenotype in murine kidney fibrosis driven by unilateral ureteral obstruction. Transcriptional profiling of IL-33-deficient kidneys 24 h after ligation revealed enhanced expression of fibrogenic genes and enrichment of gene sets involved in extracellular matrix formation and remodeling. These changes relied on intracellular effects of IL-33, because they were not reproduced by treatment with a neutralizing antibody to IL-33 that prevents IL-33R/ST2L receptor signaling nor were they observed in IL-33R/ST2-deficient kidneys. To further explore the intracellular function of IL-33, we established transcription profiles of human fibroblasts, observing that knockdown of IL-33 skewed the transcription profile from an inflammatory towards a myofibroblast phenotype, reflected in higher levels of COL3A1, COL5A1 and transgelin protein, as well as lower expression levels of *IL6*, *CXCL8*, *CLL7* and *CCL8*. In conclusion, our findings suggest that nuclear IL-33 in fibroblasts dampens the initial profibrotic response until persistent stimuli, as enforced by UUO, can override this protective mechanism.

## Introduction

Interleukin-33 (IL-33/IL1F11) is a member of the interleukin-1 superfamily that has received considerable attention as a putative mediator of fibrosis development. Much of this interest was boosted by observing that repeated injections of IL-33, acting via the transmembrane receptor ST2 (IL33R/IL1RL1), induced cutaneous fibrosis^1^ and by observing that IL-33-deficiency substantially reduced carbon tetrachloride-induced liver fibrosis^2^. Indeed, IL-33 may promote fibrosis by generating profibrotic IL-13 from type 2 innate lymphoid cells (ILC2) and T helper 2 lymphocytes^3^.

Renal interstitial fibrosis and parenchymal tubular cell loss are common end-results of chronic renal diseases and good predictors of disease progression. Current data generated from two different mouse models support the hypothesis that IL-33 also contributes to the pathology of renal fibrosis: in unilateral ureteral obstruction (UUO), Chen et al. reported reduced parenchymal loss and enhanced epithelial proliferation in both IL-33- and ST2-deficient mice^4^, and in renal ischemia–reperfusion, Liang et al. showed that inhibition of IL-33 signaling by administration of soluble IL-33 receptor (sST2) also reduced fibrosis^5^.

Reports that have addressed the fibrogenic properties of IL-33 are generally performed and interpreted in view of a mechanism by which IL-33, released from stressed or damaged cells, binds to the extracellular domain of its receptor. By contrast, sorting of IL-33 to the cell nucleus after synthesis^6–9^ and its ability to affect receptor-independent chromatin folding, suggests that IL-33 may also exert transcription factor-like, receptor-independent functions^10–12^, a mode of action that has received far less experimental attention. These considerations are all the more interesting in view of the fact that the nuclear localization of IL-33 is vital, as revealed by a lethal, non-resolving inflammation when the nuclear localization signal is disrupted^13^.

The key cellular mediator of fibrosis is the myofibroblast, which when activated serves as the primary collagen-producing and contractile cell of tissue repair. Myofibroblasts are characterized by expression of α-smooth muscle actin (α-SMA/ACTA2) that enables their contractile function and contributes to the destructive effect of fibrosis development^14,15^. We have previously demonstrated that nuclear IL-33 is rapidly and strongly induced in myofibroblasts during experimental wound healing, in gastric ulcers and in ulcerative colitis^8^. Indeed, expression of IL-33 appears to be a general feature of α-SMA-positive myofibroblasts, because it is also found in pancreatic fibrosis^16^ and in the myofibroblast-like stellate cells of the liver^17^. Taken together, these findings make it interesting to ask if IL-33 could have a role in the regulation of myofibroblast function and differentiation.

We here describe a novel role for nuclear IL-33 in regulating the myofibroblast phenotype. By means of genetic deletion or pharmacological inhibition of IL-33 in the UUO model, as well as depletion of IL-33 in activated human primary cultured dermal fibroblasts, we demonstrate that nuclear IL-33 restrains the early conversion to a full-blown myofibroblastic phenotype and supports an inflammatory phenotype. Depletion of IL-33 augmented the expression of extracellular matrix components COL1A1, COL1A2, COL3A1, COL5A1 and transgelin, but inhibited the expression of the proinflammatory cytokine IL-6 and chemokines CXCL8, CCL7 and CCL8. Our findings therefore introduce a new perspective to understanding the role of IL-33 in fibrosis development and tissue homeostasis.

## Results

### Myofibroblasts are the dominant IL-33-expressing cells in murine UUO

**Figure 1:**
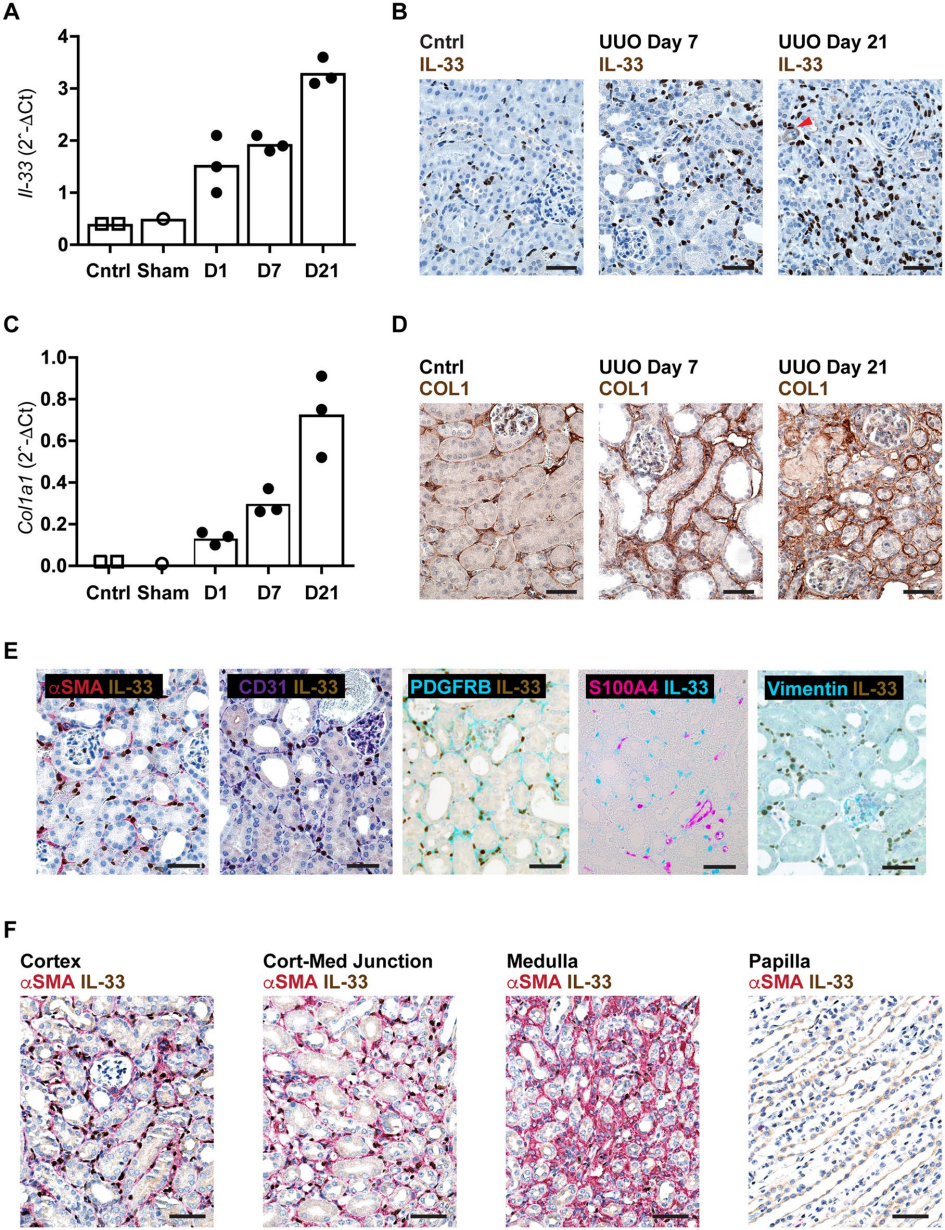
Unilateral ureteral obstruction induces IL-33 expression in mouse kidney. (**A**,**C**) Relative expression of *Il33* and *Col1a1* in kidneys from healthy control mice (n = 2) or mice subjected to sham-operation (n = 1) or UUO after 1, 7, or 21 days (n = 3 at each time point). Transcription levels were quantified as detailed in Materials and Methods by qRT-PCR. Data points represent individual mice. (**B**,**D**–**F**) Representative photomicrographs of tissue sections stained for IL-33 [brown signal in (**B**) and (**F**), brown or teal signal in (**E**)], COL1 (brown signal in panel **D)**, aSMA (red, **E**,**F**), CD31 (purple, **E**), PDGFRB (teal, **E**), Vimentin (teal, **E**) and S100A4 (purple, **E**) in kidneys from healthy control mice or mice subjected to UUO 2 days (**E**), 7 days (**B**,**D**,**F**) or 21 days (**B**,**D**) previously. Panel (**F**) shows different areas of the kidney as labelled. Scale bars = 20 μm.

A recent study by Chen and colleagues reported increased expression of IL-33 and ST2 at day 4 after unilateral ureteral obstruction^4^. We approached their experiment by collecting kidneys at 1, 7, and 21 days following UUO in C57BL/6J mice, observing a steady increase in *Il33* mRNA transcripts (Fig. 1A) and increased numbers of cells expressing nuclear IL-33 (Fig. 1B). We also observed transcription and protein expression of type I collagen that reflected the development of fibrosis (Fig. 1C,D). We then analyzed the phenotype of IL-33-expressing cells. Like Chen et al., we observed that the IL-33-expressing cell subset seen at day 2 consisted predominantly of α-SMA-positive myofibroblasts, however, we found no signal for IL-33 in CD31-positive endothelial cells nor in CD45-positive leukocytes (Fig. 1E and Figure S1). We also observed IL-33 in scattered pericytes (red arrowhead in Fig. 1A, day 21 panel). A paired immunostaining for PDGFRB, another marker for myofibroblasts and pericytes, revealed extensive colocalization (Fig. 1E) Moreover, we assessed whether IL33 was colocalized with known markers of fibroblast activation, finding that the expression of S100A4 (also known as fibroblast specific protein, FSP-1), was rather expressed in endothelial cells of capillaries and medium-sized vessels (Fig. 1E). Similarly, VIM/vimentin was also found predominantly in endothelial cells, in particular in glomeruli and medium-sized vessels, and there was no overt co-expression in IL33-positive cells (Fig. 1E). Further analysis of IL-33-expressing myofibroblasts at day 7 revealed that they were mainly localized to the cortex and the corticomedullary junction, and less abundant in the medulla and papilla (Fig. 1F).

### Genetic deletion of IL-33 has negligible impact on fibrosis progression in UUO but mediates an early boost of collagen synthesis

**Figure 2:**
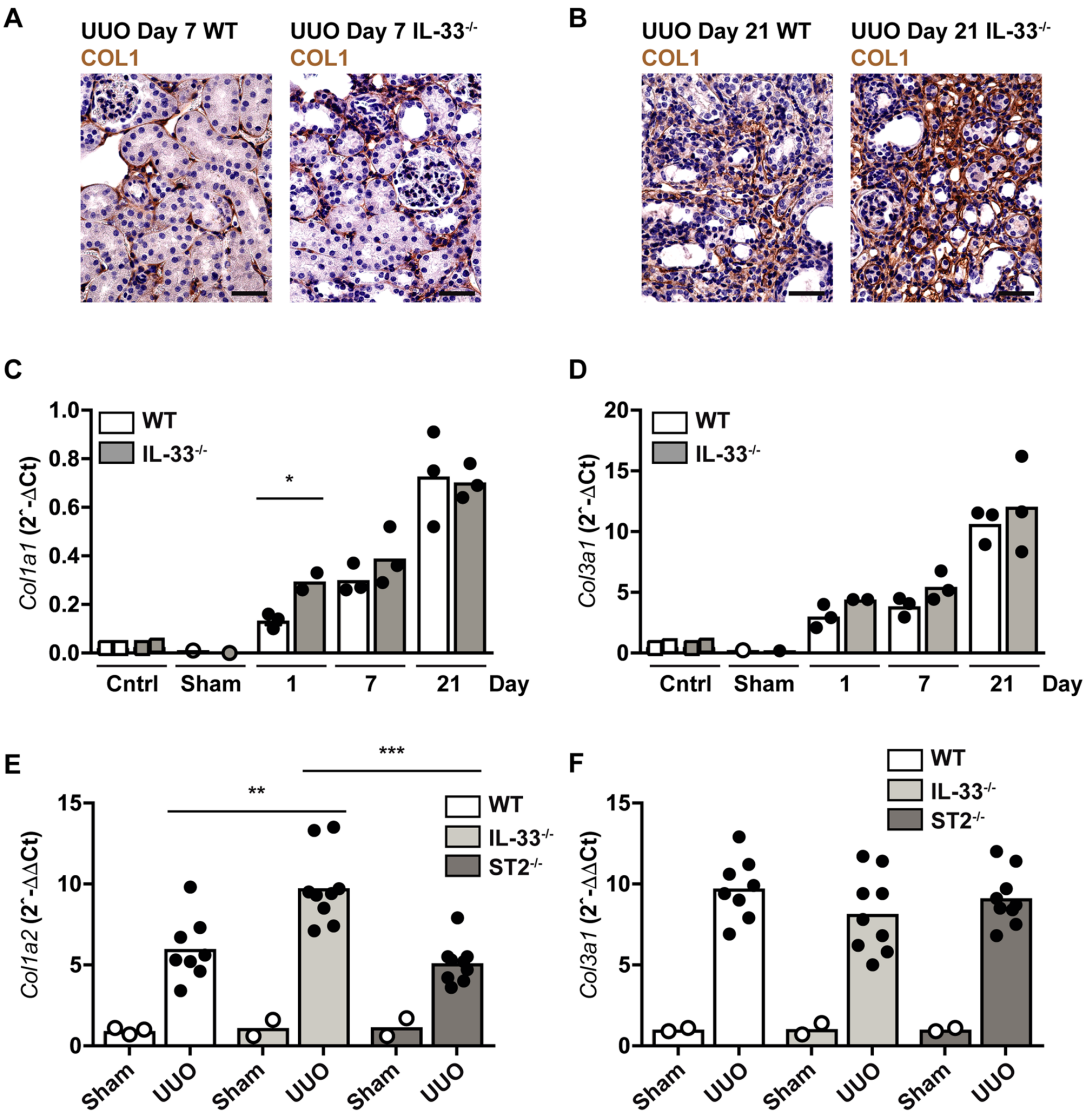
Collagen expression in IL-33^−/−^ and wildtype kidney during UUO. (**A**,**B**) Representative photomicrographs of tissue sections stained for COL1 (brown) in kidneys from IL-33^−/−^ and WT mice 7 and 21 days after UUO. (**C**,**D**) Time course comparing mRNA transcription of *Col1a1 and Col3a1* in IL-33^−/−^ and WT mice, showing healthy control mice (n = 2), sham-operated mice (n = 1) and mice subjected to UUO 1, 7, or 21 days previously (n = 3 at each time point and of each genotype). (**E**,**F**) Comparison of *Col1a1* and *Col3a1* transcription in kidneys from IL-33^−/−^, ST2^−/−^ and WT mice 4 days after sham-operation (n = 5 per genotype) or UUO (n = 10 per genotype). Transcription levels were quantified by qRT-PCR of mRNA. Data points represent individual mice, bars show mean values. *p < 0.05, **p < 0.01, ***p < 0.001.

We next assessed the putative role of IL-33 by comparing the response to renal obstruction in IL-33^−/−^ and wild type mice. In contrast to Chen and colleagues, who reported reduced fibrosis development in IL-33-deficient mice subjected to UUO^4^, we observed no difference between IL33^−/−^ and WT mice in fibrosis development at day 7 or 21 after UUO by immunohistochemical analysis of type I collagen (Fig. 2A,B). However, dissecting the fibrotic response at the transcriptional level by evaluating collagen transcripts, we observed an early, significant upregulation to two-fold higher levels of *Col1a1* transcripts and a trend to increased *Col3a1* when comparing IL-33-deficient and wildtype kidneys at day 1 (Fig. 2C,D). We therefore decided to replicate the experimental design of Chen et al. by also analyzing samples at day 4 post-UUO and including ST2-deficient mice, comparing them to IL-33-deficient and WT mice. These analyses revealed a substantial increase of *Col1a2* in IL-33-deficient kidneys compared to wildtype kidneys at day 4 but no corresponding increase in ST-2-deficient kidneys (Fig. 2E). There was no overt difference in *Col1a1* and *Col3a1* transcript levels between genotypes at day 4, indicating that the effect on these genes are shorter lasting (data not shown and Fig. 2F).

### IL-33 attenuates the expression of profibrotic genes in the early phase of UUO

**Figure 3:**
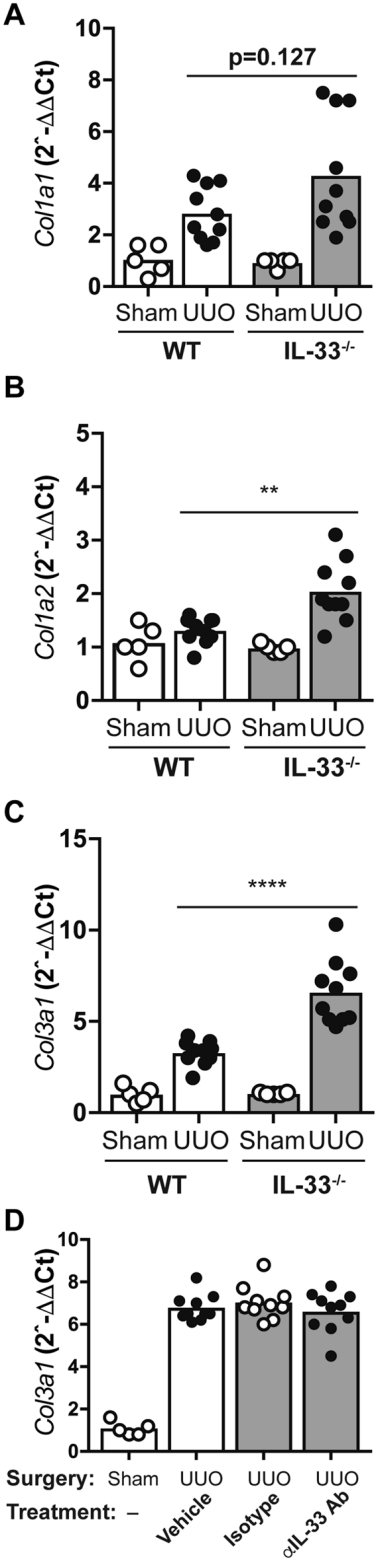
IL-33 attenuates the expression of collagens in the early phase of UUO. (**A**–**C**) Transcription of *Col1a1*, *Col1a2*, and *Col3a1* in kidneys from IL-33^−/−^ and WT mice 1 day after sham-operation (n = 5 per genotype) or UUO (n = 10 per genotype). (**D**) Transcription of *Col3a1* in kidneys from WT mice treated with anti-IL-33, isotype control antibodies or vehicle prior to UUO. Kidneys were harvested 24 h after UUO (n = 10 per treatment group) or sham-operation (n = 5). Transcription levels were quantified by qRT-PCR. Data points represent individual mice, bars show mean values. **p < 0.01, ***p < 0.001.

The increased expression of *Col1a1* and *Col31* at day 1 (Fig. 1C,D) and *Col1a2* at day 4 (Fig. 2E) in IL-33-deficient mice, indicated a role of IL-33 in the early response to injury. We explored this possibility by including more animals in an expanded experiment, observing increased transcript levels of *Col1a1, Col1a2* and *Col3a1* in IL-33-deficient mice at day 1 (Fig. 3A–C).

Having observed that IL-33 in kidney tissues from mice subjected to UUO localized to cell nuclei, we next asked if the altered gene expression observed in IL-33-deficient mice at day1 after UUO was due to the release of a repressive, nuclear IL-33 function or to the absence of extracellular IL33. We addressed the latter option by administration of a neutralizing antibody targeting IL-33 to wildtype mice before UUO (Fig. 3D). This treatment did not reproduce the fibrogenic phenotype of IL33 mutants, strengthening the hypothesis that the increase in profibrotic gene expression observed in the absence of IL-33 was due to lack of nuclear rather than extracellular IL-33.

### Transcriptome analysis of IL-33-deficient kidneys reveals profile of extracellular matrix reorganization and collagen formation

**Figure 4:**
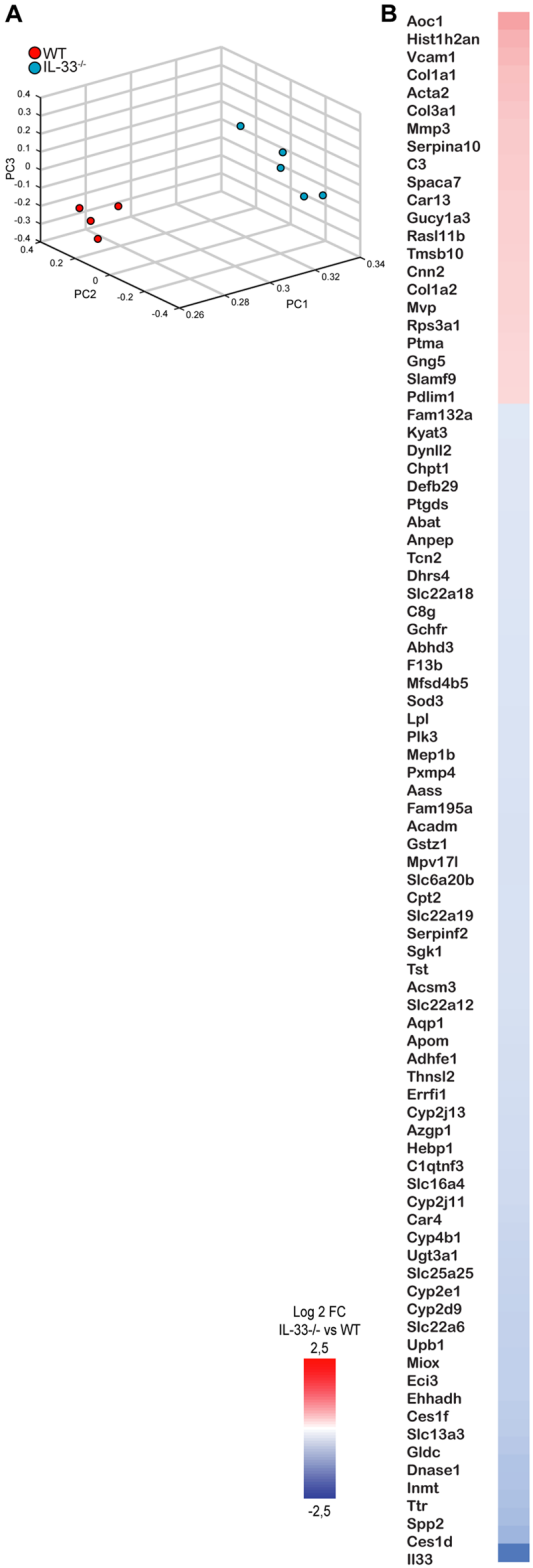
Summary of transcriptional differences between IL-33-deficient and wildtype mice 24 h after UUO. (**A**) The 3D scatter plot shows the distribution of samples based on a principal component analysis of 200 genes. (**B**) Heat map of top differentially expressed genes.

To investigate the early response to UUO in more detail by transcriptional profiling, RNA was extracted from kidneys of IL-33-deficient and wildtype mice 24 h after UUO. A pair-wise Fisher’s linear discriminant^24^ was used to identify the top 5% (200 genes) differentially expressed genes between the WT and IL-33-deficient kidneys revealing a clear separation between genotypes in a principal component analysis (Fig. 4A). A non-stringent approach (p < 0.05, fdr < 0.25, and fold change > 1.3) identified 134 downregulated and 46 upregulated probes in the contrast IL33^−/−^ (n = 5) versus WT (n = 4) (Suppl. Table S1), among the latter notably *Col1a1*, *Col1a12* and *Col3a1* as well as myofibroblast-associated *Acta2* (Fig. 4B). In addition, gene ontology analysis of differentially expressed genes using DAVID annotation clustering (Table S2) revealed a positive enrichment for GO-terms associated with collagens and protease inhibitors, as well as PDGF binding and ECM-receptor interaction, and negative enrichment for GO-terms associated with oxidoreductase activity and sodium-independent anion transporters.

### Depletion of fibroblast IL-33 promotes a gene signature consistent with increased extracellular matrix deposition

**Figure 5:**
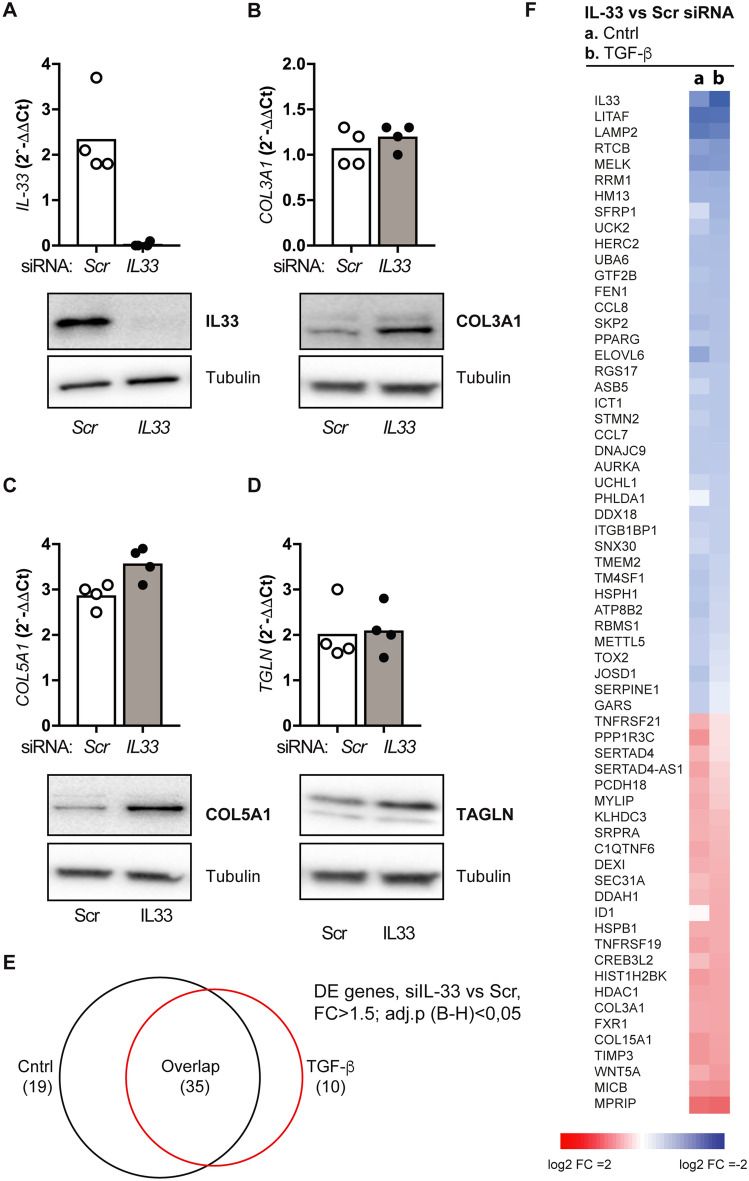
Depletion of fibroblast IL-33 enforces a gene signature consistent with increased extracellular matrix deposition. Expression of *IL33* (**A**), *COL5A1* (**B**)*, FIBL5* (**C**) and *TGLN* (**D**) expression in human fibroblasts treated with IL-33-specific and scrambled siRNA was measured by qRT-PCR (top panels) and western blot (bottom panels). Bars show mean values of 4 independent experiments, *p < 0.05. (**E**) Venn diagram comparing differential expression of transcripts in response to silencing of IL33 between control and activated cells. (**F**) Heat map showing differentially expressed transcripts in response to siRNA-silencing of IL-33 in control and activated cells.

To explore the function of IL-33 in the dominant IL-33-expressing cell population of obstructed kidneys, we skewed cultured human fibroblasts towards a myofibroblast phenotype by exposing them to IFN-γ and TGF-β for 24 h^8^ after siRNA-mediated knockdown of IL-33. Efficient knockdown was verified both at the protein and transcript levels (Fig. 5A) and was accompanied by increased mRNA and protein levels of profibrotic COL3A1, COL5A1 and TAGLN (Fig. 5B–D). Initial analyses indicated an involvement of TGF-β and prompted us to transcriptionally profile pooled, cultured human fibroblasts treated with siRNA to IL-33 and activated by IFN-γ (to induce IL-33 expression) or by the combination of IFN-γ and TGF-β for 24 h. This analysis identified 64 differentially expressed genes associated with depletion of IL-33 (adj.p < 0.05 and fold change > 1.5, Fig. 5E), 35 of which were common to both cytokine treatments (Fig. 5E,F). Furthermore, most of the genes showed similar trends under both conditions, even if they reached the chosen threshold for differential expression only under one of them (Fig. 5F), supporting the assumption that the effect of inhibiting IL-33 was similar under both conditions. For gene ontology analysis, we therefore considered genes that were differentially expressed by IL-33 depletion under at least one condition and performed annotation clustering (Table S3). In agreement with our finding in UUO-exposed, IL-33-deficient kidneys, we found that inhibition of IL-33 in fibroblasts led to increased expression of genes enriched for GO-terms associated with collagen and extracellular matrix components. Furthermore, genes repressed by depletion of IL-33 were enriched for GO-terms associated with a response to inflammatory stimulation. Together, our data imply that IL-33 expression in fibroblasts serves to dampen the upregulation of transcripts related to extracellular matrix deposition and myofibroblast activation, while inflammatory transcripts to some extent are supported by IL-33.

### Depletion of IL-33 in activated fibroblasts favors a myofibroblastic over an inflammatory phenotype

**Figure 6:**
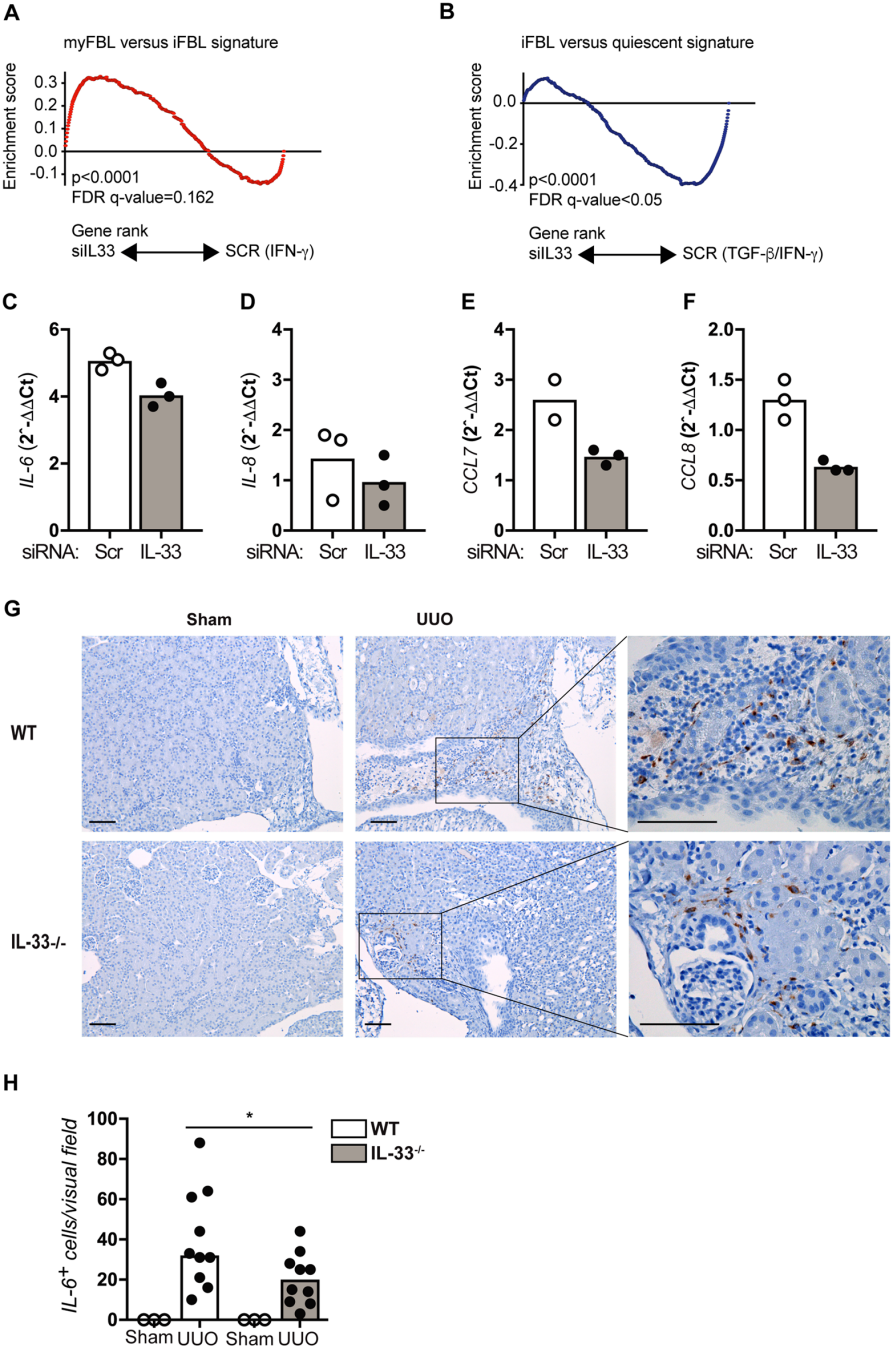
Depletion of IL-33 skews fibroblasts towards a myofibroblastic phenotype. (**A**,**B**) Gene set enrichment analysis showed that cells treated with IL-33-specific siRNA were positively enriched for a myofibroblast (myFBL) versus inflammatory fibroblast (iFBL) signature and negatively enriched for an inflammatory versus quiescent fibroblast signature. (**C**–**F**) Transcription of *IL6* (**C**), *IL8* (**D**)*, CCL7* (**E**) and *CCL8* (**F**) in human fibroblasts treated with IL33-specific or scrambled siRNA was measured by qRT-PCR. Each data point shows mean values of technical triplicates from independent experiments. (**G**) Representative photomicrographs of kidney sections harvested 4 days after sham or UUO surgery from WT and IL-33^−/−^ mice and immunostained for IL-6 (brown signal). Scale bars = 100 μm. (**H**) Blinded quantification of signal enumerating the average number of positive cells per high-power visual field. Individual data points show the average number of positive cells in one mouse, counting 3 high power fields in areas of highest density. *p < 0.05.

Based on our observation that the effect of IL-33 appears to persist and get more prominent over time even though IL-33 expression declines (Figure S2A,B), we next asked if the main nuclear function of IL-33 might be the regulation of fibroblast differentiation rather than a direct regulation of pro-fibrotic effectors. Upon stimulation, quiescent fibroblasts and other mesenchymal cells develop inflammatory and/or contractile functions. The spectrum between inflammatory fibroblasts and contractile myofibroblasts remains poorly defined and probably represents a sliding transition rather than two distinct phenotypes^14^. However, a recent publication by Öhlund et al.^23^ compared cancer-associated fibroblasts (CAFs) to quiescent pancreatic stellate cells, identifying an inflammatory phenotype termed iCAFs (co-cultured in Transwells with cytokine-secreting tumor organoids and expressing high and low levels of IL6 and SMA, respectively [IL6^high^α-SMA^low^]) and a contractile myofibroblastic phenotype, myCAFs (IL6^low^α-SMA^high^, cultured in dense monolayers). Transcriptional profiling and comparison of iCAFs and myCAFs identified characteristic transcriptional profiles. While fibroblasts with an inflammatory phenotype (iCAFs) boosted their expression of interleukins and chemokines, those with a myofibroblastic phenotype (myCAFs) were characterized by enhanced expression of TGF-β response genes and extracellular matrix components including collagens^23^. We used this data set to generate signature lists consisting of the top differentially regulated genes between phenotypes, and performed gene set enrichment analysis in the GSEA software^22^. Results indicated that inhibition of IL-33 supported a contractile myofibroblastic phenotype (myFBL) and inhibited an inflammatory phenotype (iFBL) in human fibroblasts (Fig. 6A,B, Table S4), thus agreeing with results from our gene ontology analysis. At this point, we turned our attention to *IL6* and *CXCL8*, cytokines that have been proposed as markers for iCAFs^23^, finding that although these genes did not fulfill criteria for differential expression in our transcriptome analysis, a reduction in their expression levels could be detected by qRT-PCR following depletion of IL-33 (Fig. 6C,D). Among inflammatory mediators found to be downregulated in our transcriptome analysis (Fig. 5F), we also confirmed a reduction in expression for *CCL7* and *CCL8* in cells treated with anti-IL-33 siRNA (Fig. 6E,F).

These observations prompted us to investigate if our findings could be translated to the murine UUO model at day 1. Immunohistochemical analysis (Fig. 6G) and quantification (Fig. 6H) revealed the induction of IL-6-expressing cells in wildtype kidneys, similar to observations on the transcript level by Chen et al.^4^. By contrast, we observed significantly reduced numbers of IL-6-positive cells in IL-33^−/−^ mice, in good agreement with our in vitro-generated human fibroblast data.

## Discussion

Since its discovery, a broad impact of tissue-derived IL-33 has been demonstrated in cardiac hypertrophic remodeling^25^, and in fibrosis of lung^26^, skin^1^, and liver^2^. These effects are all mediated through interactions with its cognate membrane-bound receptor ST2 (IL33R/IL1RL1). Far less attention has been paid to the potential of this intranuclear cytokine to modulate transcription^10^ in tissue-resident stromal cells, and the functional significance of nuclear IL-33 remains largely unknown^27^. Here, we combined analyses of unilateral ureteral obstruction in vivo with analyses of human fibroblasts in vitro to reveal a novel role for nuclear IL-33 as a repressor of interstitial cell extracellular matrix deposition. This conclusion was drawn from observing that early pro-fibrotic responses were amplified in IL-33-deficient mice but not by neutralizing IL-33 antibodies given to wild-type mice and observing that depletion of IL-33 in human dermal fibroblasts augmented the expression of extracellular matrix components and skewed activated fibroblasts towards a prototypic myofibroblastic phenotype.

A nuclear localization of IL-33 was observed both in cells in the renal interstitial space that as a sequela to ureteral obstruction develop into α-SMA + myofibroblasts, and in primary human fibroblasts exposed to profibrotic stimuli. Moreover, the increased collagen transcription observed in IL-33-deficient mice, was seen neither in wild-type mice given neutralizing IL-33 antibodies nor in ST2-deficient mice, indicating that IL-33 dampens the expression of extracellular matrix components through its nuclear functions. We therefore propose that IL-33 exerts active homeostatic nuclear functions in fibroblasts that protect the host from unwarranted responses. In this manner, IL-33 induced by the inflammatory environment may act as a natural suppressor of profibrotic genes, appearing to be accelerated by the presence of pro-fibrotic cytokines like TGF-β. This model correlates well with the recent observation that nuclear, but not extracellular, IL-33 exerts beneficial effects on wound healing in mice, by suppressing NFkB activity in keratinocytes and limiting excessive inflammation^28^.

The early increase in collagens and extracellular matrix genes did not translate to increased fibrosis development in the UUO model. A likely explanation for this apparent discrepancy could be that the unrelenting pressure and subsequent tissue-damage induced by complete ureteral obstruction is strong enough to override the homeostatic mechanism exerted by IL-33. Considering the irreversible nature of UUO and its ensuing impact on renal function, this is perhaps not surprising. One should also consider that the cellular stress and damage exerted by UUO at later time points could mediate the release of IL-33 to the extracellular space^7,29^, reducing its accumulation in the nucleus and, perhaps more importantly, enabling pro-fibrotic signaling via its receptor ST2L. Indeed, a pro-fibrotic role for IL-33-ST2L signaling has been reported in many organs, including the kidney^4,5^. However, we did not observe any evidence of a pro-fibrotic role for IL-33 in our system even in efforts to reproduce the exact experimental conditions previously used to demonstrate this phenomenon.

Nuclear expression of IL-33 in fibroblasts is induced by inflammatory activation^8,30^, and wound-healing experiments revealed that this induction is associated with an early activation state of the cell^8^. The effect of nuclear IL-33 is therefore unlikely to be exerted on the quiescent fibroblast, but rather at an intermediate stage of activation. Our observation that depletion of IL-33 augmented the expression of extracellular matrix components like COL5A1 and TGLN, but reduced the expression of proinflammatory cytokine *IL6* and chemokines *CXCL8, CCL7* and *CCL8*, indicates that the model proposing a distinction between inflammatory (IL6^high^α-SMA^low^) and myofibroblastic (IL6^low^α-SMA^high^) fibroblastic phenotypes in pancreatic cancer^23^ could be highly relevant also when considering the nuclear function of IL-33 in cultured myofibroblasts. Potential effects on tissue inflammation should be kept in mind when considering the overall effect of IL-33-deficiency on fibrosis development. Inflammation is a major drive for fibrosis, and, at least in the lung, fibroblast secretion of inflammatory cytokines like CCL8 contributes to fibrosis pathology^31^. One can therefore not automatically assume that a bias towards the inflammatory fibroblastic phenotype inhibits long term development of fibrosis.

Our findings from cell culture indicate that, over time, fully converted myofibroblasts may downregulate nuclear IL-33. Although IL-33 is downregulated in fibroblast cultures with prolonged exposure to TGF-β, the effect of IL-33-depletion persists, most likely due to a higher proportion of fully differentiated myofibroblasts in the culture. This observation also supports the hypothesis that the effect of IL-33 is due to a modulation of phenotype conversion rather than through direct intracellular interactions that inhibit pro-fibrotic signaling.

Future experiments should be designed to investigate the impact of nuclear IL-33 function in fibroblasts under different conditions and to identify the molecular mechanisms involved. A very interesting approach would be to map the chromatin regions occupied by IL-33 in activated fibroblasts by chromatin immunoprecipitation followed by deep sequencing and to identify interaction partners by immunoprecipitation and mass spectrometry. Another approach would be single-cell transcriptional profiling. In this context it also deserves mention that our murine in vivo data were supplemented by in vitro experiments in human fibroblasts. As primary cell cultures from human kidney have not been easily available to us, we prioritized relevance to *species of interest* over relevance to *organ of interest* in choosing to explore and extend our in vivo data in a mechanistic in vitro model.

Our combined findings make it tempting to propose a model where early tissue damage-induced activation of mesenchymal/fibroblastic cells induces an intermediate fibroblastic phenotype with high expression of nuclear IL-33 and predominantly inflammatory features. In the absence of further tissue damage, this nuclear IL-33 may function to inhibit the full-blown conversion to an extracellular matrix-producing, myofibroblastic phenotype, thereby preventing excessive scar formation and preserving organ function. However, if the damaging stimulus persists, as in UUO, the protective functions of IL-33 are overridden, and IL-33 may even be secreted from activated fibroblasts^29^ to exert pro-fibrotic actions through activation of ST2. In this context, a pharmacological intervention aiming at targeting extracellular IL-33, and therefore blocking the pro-inflammatory and pro-fibrotic signal of the IL-33–ST2L axis, might be beneficial in the treatment of inflammatory and fibrotic diseases.

## Materials and methods

### Unilateral ureteral obstruction (UUO)

*Il33*- and *ilrl1-*deficient mice were obtained from Merck Research Laboratories and Stefan Wirtz’ laboratory, respectively. Both strains were backcrossed to the Taconic C57BL/6J BomTac (B6JBOM) background for ten generations to ensure genetic homogeneity. All experiments involving animals were performed according to institutional guidelines, following protocols approved by the Norwegian Food Safety Authority (FOTS 6997). Unless differently stated, the mice used for experiments were 8 weeks old, n = 10 in the UUO group and n = 5 in the sham-operated group. The UUO procedure was performed under isofluorane/O_2_ anesthesia by making a small incision in the left flank. The kidney was gently retracted from the retroabdominal space and one metal clip (MCS-20 Ligaclip Ethicon) applied to obstruct the ureter. The kidney was then gently relocated and the wound sutured. Animals were treated with buprenorphine (0.05 mg/kg) 30 min before surgery as premedication, and every 12 h for the following 48 h as analgesia. Neutralization of IL-33 in WT mice was performed by injecting the rat anti-mouse IL-33 (clone M19, IgG2a, 200 µg per mouse i.p. 24 h before UUO) and using rat anti-human creatine kinase (clone CHO-CK, IgG2a) as an isotype control. Vehicle-treated (PBS) animals were also used as controls.

### Fibroblast culture

Primary normal human dermal fibroblasts (NHDF) were purchased from PromoCell (Heidelberg, Germany) and cultured in Fibroblast Growth Medium supplemented with one vial of SupplementMix (PromoCell), 5% FCS, 100 μg/ml amphotericin B and 100 μg/ml gentamicin (Thermo Fisher Scientific). Cells were used at passage level three to five, maintained at 37 °C in 95% humidity/5% CO_2_ atmosphere and split at a ratio not exceeding 1:3. A minimum of cells from 2 different donors were pooled to reduce biological variation.

### Gene silencing

Human IL-33 (#4392422) and Silencer Select Negative Control No. 2 siRNA (#4390846**)** were purchased from Thermo Fisher Scientific (Oslo, Norway). Fibroblasts were transfected with 30 nM siRNA using Lipofectamine RNAimax (Invitrogen, Oslo) in Opti-MEM for 12 h followed by a change to serum free fibroblast growth medium, containing IFN-γ (100 ng/ml) and TGF-β (2 ng/ml) for another 12 h before harvest and RNA extraction.

### RNA extraction and qRT-PCR

Harvested tissue was stabilized in RNAlater (Life Technologies, Carlsbad, CA) and stored at − 80 °C. Samples were homogenized in RLT buffer from the RNeasy Mini Kit (Qiagen, Norway, Oslo) with 6.35 mm diameter ceramic spheres (MP Biomedicals, Santa Ana, CA) in a bead-milling homogenator (MP Biomedicals FastPrep-24). RNA from these homogenates or from cell cultures, was isolated with the RNeasy Mini Kit according to the manufacturer’s instructions (Qiagen, Oslo, Norway). RNA purification was performed using an RNase-Free DNAse Set (Qiagen, Oslo, Norway). RNA concentration and quality were assessed with an ND1000 NanoDrop Spectrophotometer (ThermoFisher). Reverse transcription of 1 μg of RNA was performed with SuperScriptIII Reverse Transcriptase cDNA system (Invitrogen), oligo (dT) primers, and dNTPs (GE Healthcare, Oslo), according to manufacturer’s instructions. The PCR reaction consisted of 5 μl of 10-times diluted cDNA in a 20-μl qPCR reaction consisting of 5000 U Hot-Start Taq DNA polymerase (Qiagen, Oslo, Norway), dNTP (GE Healthcare), 20 × EvaGreen (Biotium), and the individual primer sets (Table 1). Quantitative RT-PCR was performed with the Stratagene Mx3005P instrument and Stratagene MxPro software (Agilent Technologies, Santa Clara, CA) with the following program: hot start at 95 °C for 15 min, amplification 40 cycles at 95 °C for 30 s, 60 °C for 30 s, 72 °C for 30 s, melting 95 °C for 1 min, 57 °C for 30 s, 95 °C for 30 s, 40 °C for 10 s. Data were analyzed according to the comparative C_T_ method (2^−ΔΔCT^ method) of Schmittgen and Livak^18^, where the internal control relative to constitutive gene expression was provided by *hprt* gene expression (2^−ΔCT^), and where indicated, these results was further normalized to an experimental control (2^−ΔΔCT^), in UUO experiments by comparison to sham-operated mice and in cell cultures using untreated cells.

**Table 1:** Primers used for RT-PCR.

Gene target	Forward primer sequence	Reverse primer sequence
Il33*	AGGCGACGGTGTGGATGGGA	CGTCACCCCTTTGAAGCTCCACG
*Col1a2*	CCGTGCTTCTCAGAACATCA	ACCAGAATCTGTCCACCAGTGCTT
*Col3a1*	TCCTAACCAAGGCTGCAAGATGGA	ACCAGAATCTGTCCACCAGTGCTT
*Acta2*	ATTGTGCTGGACTCTGGAGATGGT	TGATGTCACGGACAATCTCACGCT
*Hprt*	TGATCAGTCAACGGGGGACA	TTCGAGAGGTCCTTTTCACCA
IL33**	CCACAGCAAAGTGGAAGAACACAGCA	GGTGGTTTCTCTCCTAAAGTAACAGGCCT
*COL3A1*	GCTCTGCTTCATCCCACTATTA	CTGGCTTCCAGACATCTCTATC
*COL5A1*	GACTGCCAGATTTGGACACTAT	GGATGACCTTTACGAGGCTTAC
*FBL5*	CCACTCTCAGCTCCAAACTATC	CTCGTCCACATCCACACATT
*TAGLN*	CCAGACTGTTGACCTCTTTGA	CGGTAGTGCCCATCATTCTT
*ACTA2*	GACCGAATGCAGAAGGAGAT	CACCGATCCAGACAGAGTATTT
*HPRT*	AATACAAAGCCTAAGATGAGAGTTCAAGTTGAGTT	CTATAGGCTCATAGTGCAAATAAACAGTTTAGGAAT

*Italicized lower-case characters for mouse genes.

**Italicized upper-case characters for human genes.

### Gene expression profiling

Total RNA (500 ng) from each sample of UUO tissue or cultured fibroblasts was amplified and labeled using the Illumina Total Prep 96 RNA Amp Kit. The quality and size distribution of labeled cRNA was assessed using the NanoDrop Spectrophotometer and the 2100 Bioanalyzer (Agilent), before 750 ng of biotin-labeled cRNA was hybridized to the Illumina MouseRef-8 v2 Expression BeadChip or the HumanHT-12 v4 Expression BeadChip. Microarrays were performed at Norwegian Genomics Consortium in Oslo, Norway. The microarray data sets have been deposited as a super series in National Center for Biotechnology Information GEO (Gene Expression Omnibus) and are accessible through the accession number GSE149918.

Array data were first imported to R/Bioconductor (RCoreTeam, 2016) using the beadarray package^19^, and differentially expressed genes were identified using the limma package^20^. Principal component analysis (PCA) and 3-D PCA plots were performed in MATLAB statistics toolbox. Gene ontology analysis was performed using functional annotation clustering in DAVID^21^. Gene Set enrichment analysis was performed using the GSEA software^22^ and the HALLMARK gene set collection provided by the Molecular Signature Database (http://www.broad.mit.edu/gsea/). The GSEA signatures used for analysis of the fibroblast phenotypes were custom made from a recently published dataset^23^ and consisted of the top differentially expressed genes between myofibroblastic and inflammatory (500 genes), myofibroblastic and quiescent (350 genes), inflammatory and myofibroblastic (500 genes), and inflammatory and quiescent (500 genes) phenotypes.

### Immunohisto/cytochemistry

Tissue samples were fixed overnight in formalin 10%, washed in phosphate buffered saline (PBS), dehydrated and embedded in paraffin. Tissue sections were deparaffinized with xylene, rehydrated in graded alcohol and antigen-retrieved by boiling in 10 mM Tris–EDTA Target Retrieval solution (pH 9, 20 min; Dako-Agilent, Santa Clara, CA). Pretreatment was made by incubating the sections with Peroxidase Blocking solution (5 min at RT, Dako-Agilent, Santa Clara, CA). Primary antibodies to CD31 (RB10333, Thermo), COL1 (Ab34710, Abcam), IL-33 (AD3626, R&D Systems), ACTA2 (M0851, Dako) or IL-6 (D5W4V, Cell Signaling, Danvers, MA) were diluted in Discovery Ab Diluent (Ventana Medical Systems-Roche, CA) and incubated 1 h at RT followed by incubation with secondary antibody according to detection kit’s instructions: Mouse-on-Mouse HRP-Polymer (10 min at RT), or Rabbit-on-Rodent HRP-Polymer (30 min at RT) or Goat-on-Rodent HRP-Polymer Detection kit (Biocare Medical, Pacheco, CA) and subsequently incubation with EnVision Flex DAB + Chromogen (5 min at RT; Dako-Agilent, Santa Clara, CA). Specimens were counterstained in Shandon Instant Hematoxylin (30 s, Thermo Scientific, UK), dehydrated and mounted in Pertex mounting medium (HistoLab, Norway).

IL-6-positive cells were enumerated by selecting 3 high-power visual fields with the highest density in each animal and plotting average count per high-power field. Enumeration was performed by one blinded observer (CH).

### Western blot analysis

Cultured fibroblasts were washed in PBS and lysed in Tris–HCl buffer (pH 6.8) containing SDS (2.5%), glycerol (10%), 100 mM 2-mercaptoethanol (Sigma, St Louis, MO), Protease Inhibitor Cocktail (Roche) and phosphatase inhibitor sodium orthovanadate (2 nM, Sigma-Aldrich). Samples were passed through a QIAshredder column (Qiagen) at 10,000 rpm for 5 min, boiled for 5 min and separated on a 10% or a 4–20% Mini-PROTEAN TGX Precast Gel (both from Bio-Rad) depending on the molecular weight of target proteins. Gels were run for 15–25 min at 300 V before blotting to nitrocellulose membranes using the Trans-Blot Turbo Transfer System and Turbo blotter (both from Bio-Rad) and blocking with 5% Blotting-Grade Blocker (Bio-Rad). Primary antibodies to COL3A1 (NB600-594, NovusBio), COL5A1 (NBP1-19633, NovusBio), IL33 (Alx-804-840-0000, Enzo), TAGLN (Ab14106, Abcam) or TUBB (loading control, Ab6046, Abcam) were incubated over night at 4 °C. After the incubation of appropriate secondary horseradish peroxidase-conjugated antibody for 2 h at RT, the protein bands on the membrane were detected with Pierce SuperSignal West Dura Extended Duration Substrate (Thermo Fischer Scientific) and visualized using a ChemiDoc MP System (Bio-Rad).

### Statistics

Data from both in vivo and in vitro experiments were analyzed with Prism v7 (GraphPad), using the Mann–Whitney *U* non-parametric test to analyze differences between groups or *t* test if n > 5. P values lower than 0.05 were considered statistically significant. Data from UUO experiments show values from single animals (plotted as mean values of technical duplicates) and the mean ± SEM of each experimental group. In vitro experiments are shown with mean values of technical triplicates and mean ± SEM of independent experiments.

## Supplementary Information

Supplementary Information.

## Data Availability

The microarray data sets have been deposited as a super series in National Center for Biotechnology Information GEO (Gene Expression Omnibus) and are accessible through the accession number GSE149918.
