# Supplementary material for: Nuclear IL-33 restrains the early conversion of fibroblasts to an extracellular matrix-secreting phenotype
Source: Sci Rep. 2021 Jan 8;11:108. doi: 10.1038/s41598-020-80509-5 (PMC7794291; doi:10.1038/s41598-020-80509-5)
Supplement: Supplementary file 1 — Supplementary Information. [file 41598_2020_80509_MOESM1_ESM.pdf]

Nuclear IL-33 restrains the early conversion of fibroblasts to an extracellular matrix-secreting phenotype

Francesca Gatti[1,2#], Sobuj Mia[1,2#], Clara Hammarström[1,2], Nadine Frerker[1,2], Bjarte Fosby[3], Junbai Wang[1], Wojciech Pietka[1,2], Olav Sundnes[1,2], Johanna Hol[1,2], Monika Kasprzycka[1,2], and Guttorm Haraldsen[1,2*]

[1]Department of Pathology, [2]K.G. Jebsen Inflammation Research Centre, [3]Dept of Surgery, University of Oslo and Oslo University Hospital, Rikshospitalet, Norway.

[#]*These authors contributed equally to the study.*
**Corresponding author*

Number of figures: 7

Number of tables: 5

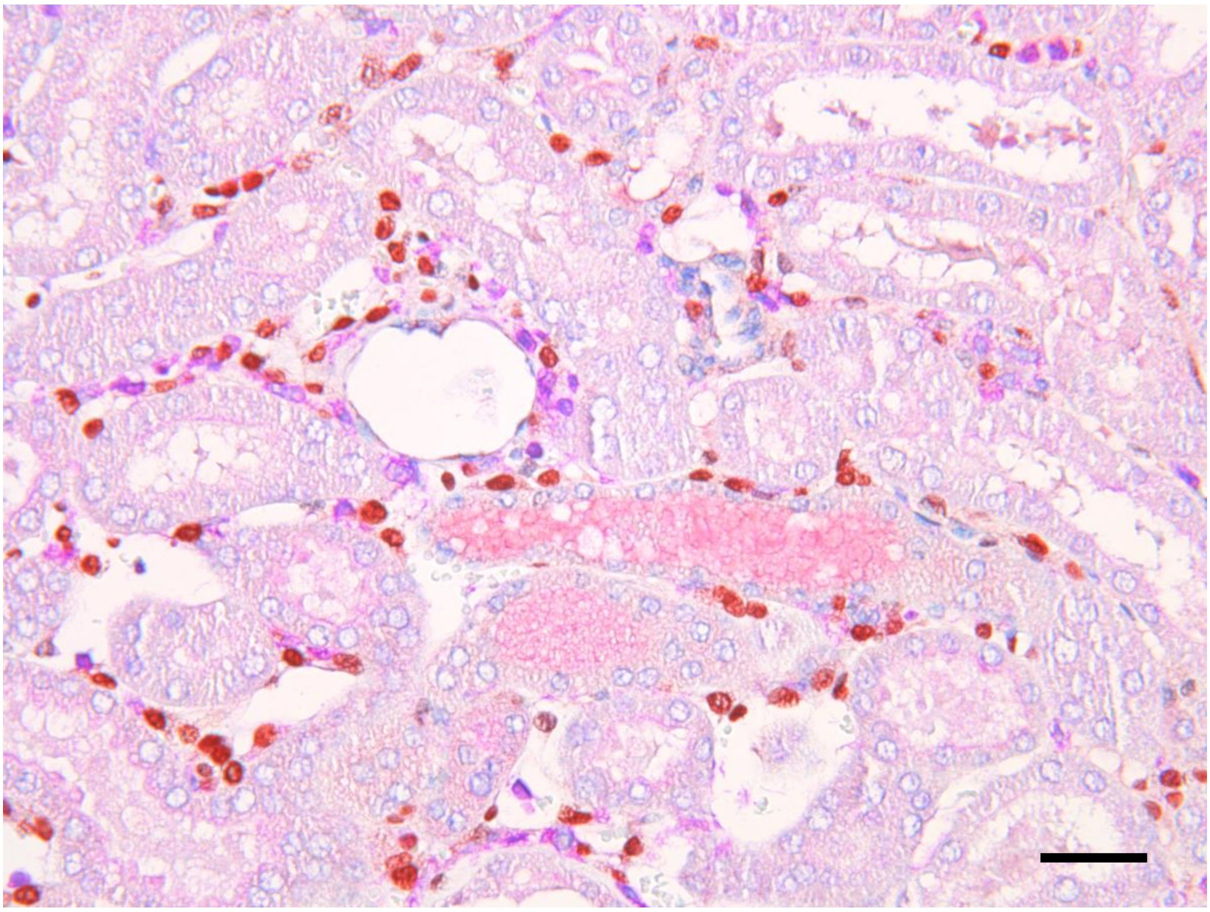

Figure S1. Expression of IL-33 and CD45 in day 2 post-UUO of mouse kidney.
Representative photomicrograph of tissue sections stained for IL-33 (brown signal) and CD45 (purple) in kidney from a mouse subjected to UUO 2 days. Scale bar = 50µm.

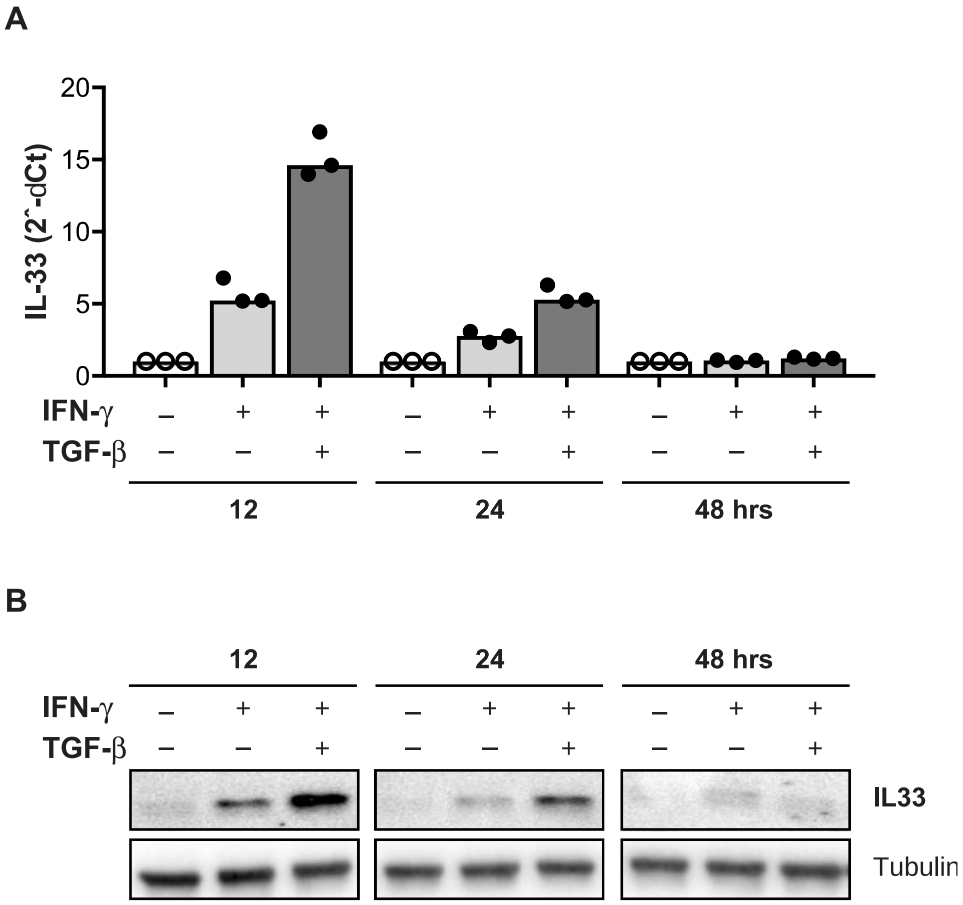

Figure S2. Expression of IL-33 in cultured human fibroblasts. IL-33 mRNA (A) and protein (B) expression was measured after 12, 24, and 48 h exposure to IFN-γ alone or in combination with TGF-β, by RT-qPCR or western blot, respectively. Individual data point (A) show mean values of technical triplicates from 3 independent experiments, whereas (B) shows one representative immunoblots of at least 3 independent experiments.

Gatti et al - Figure S3

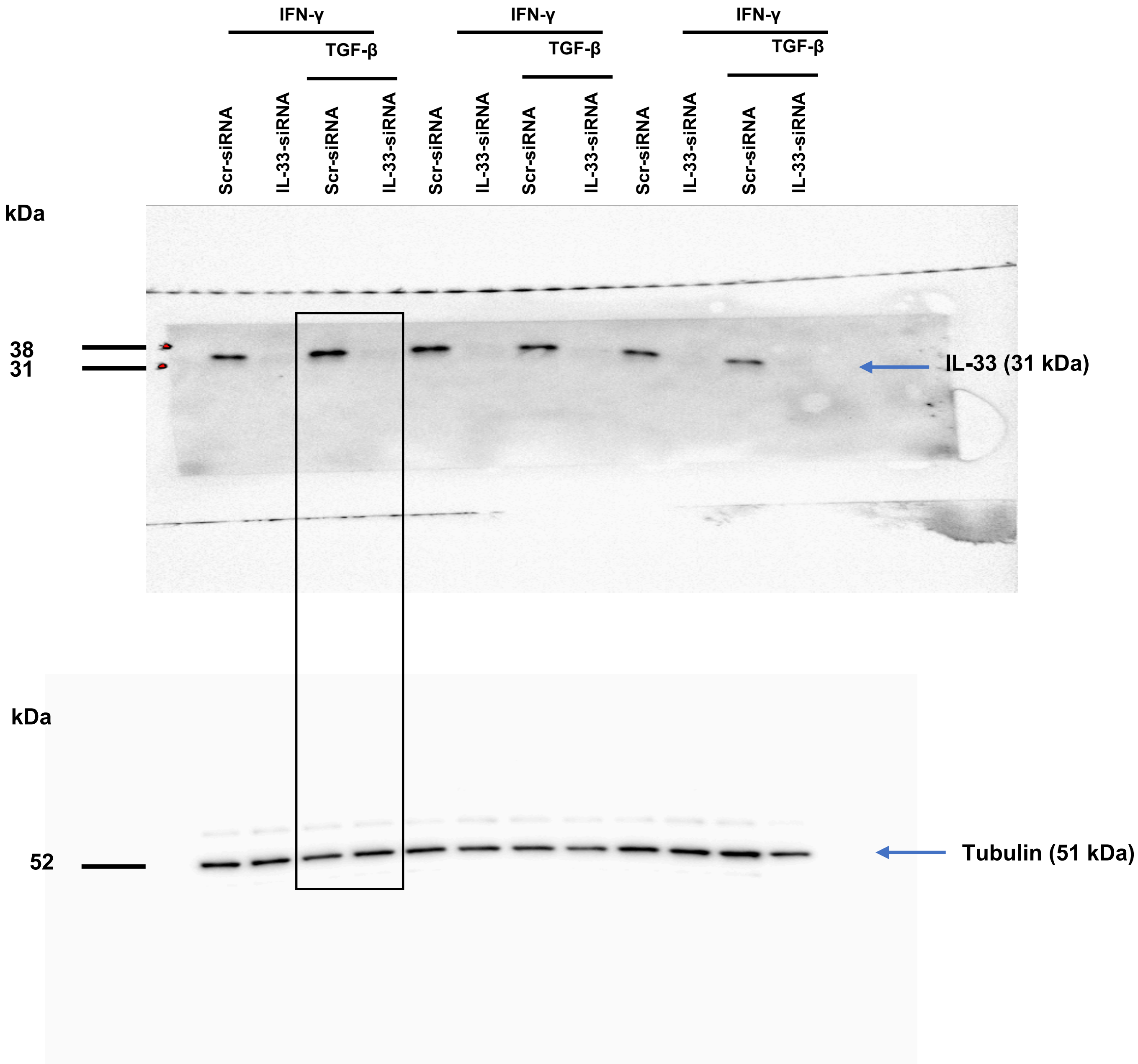

Gatti et al - Figure S4

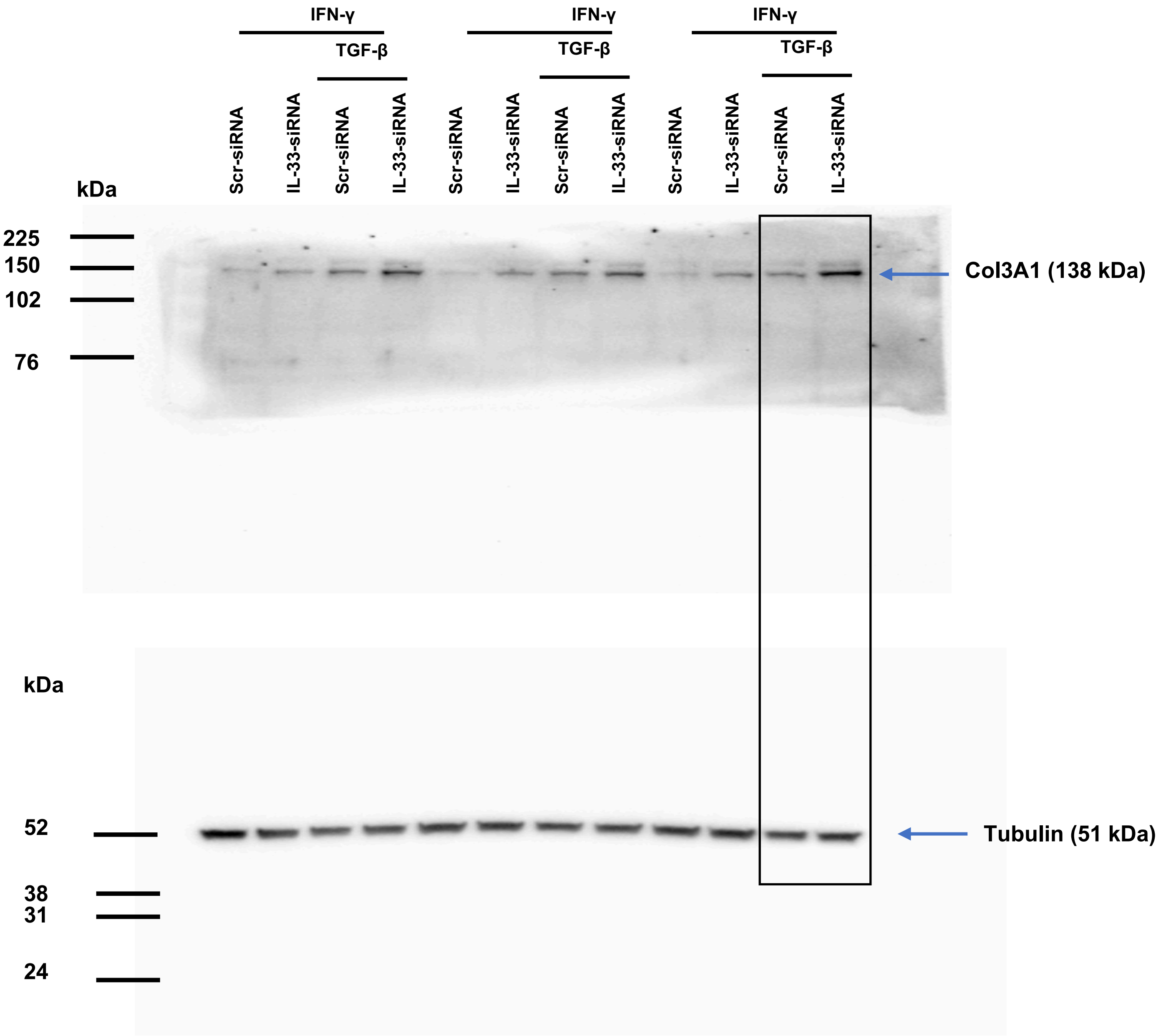

Gatti et al - Figure S5

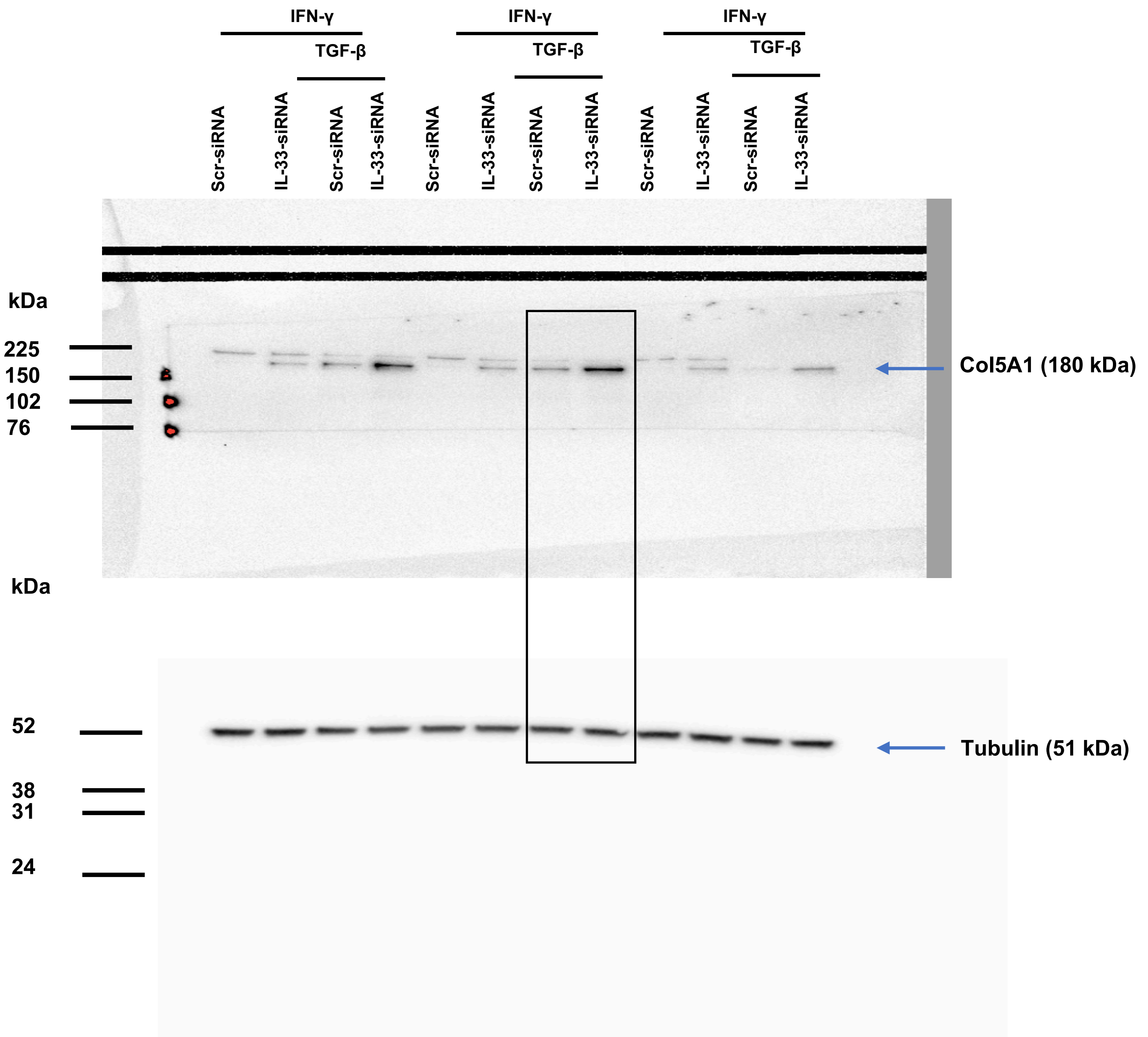

Gatti et al - Figure S6

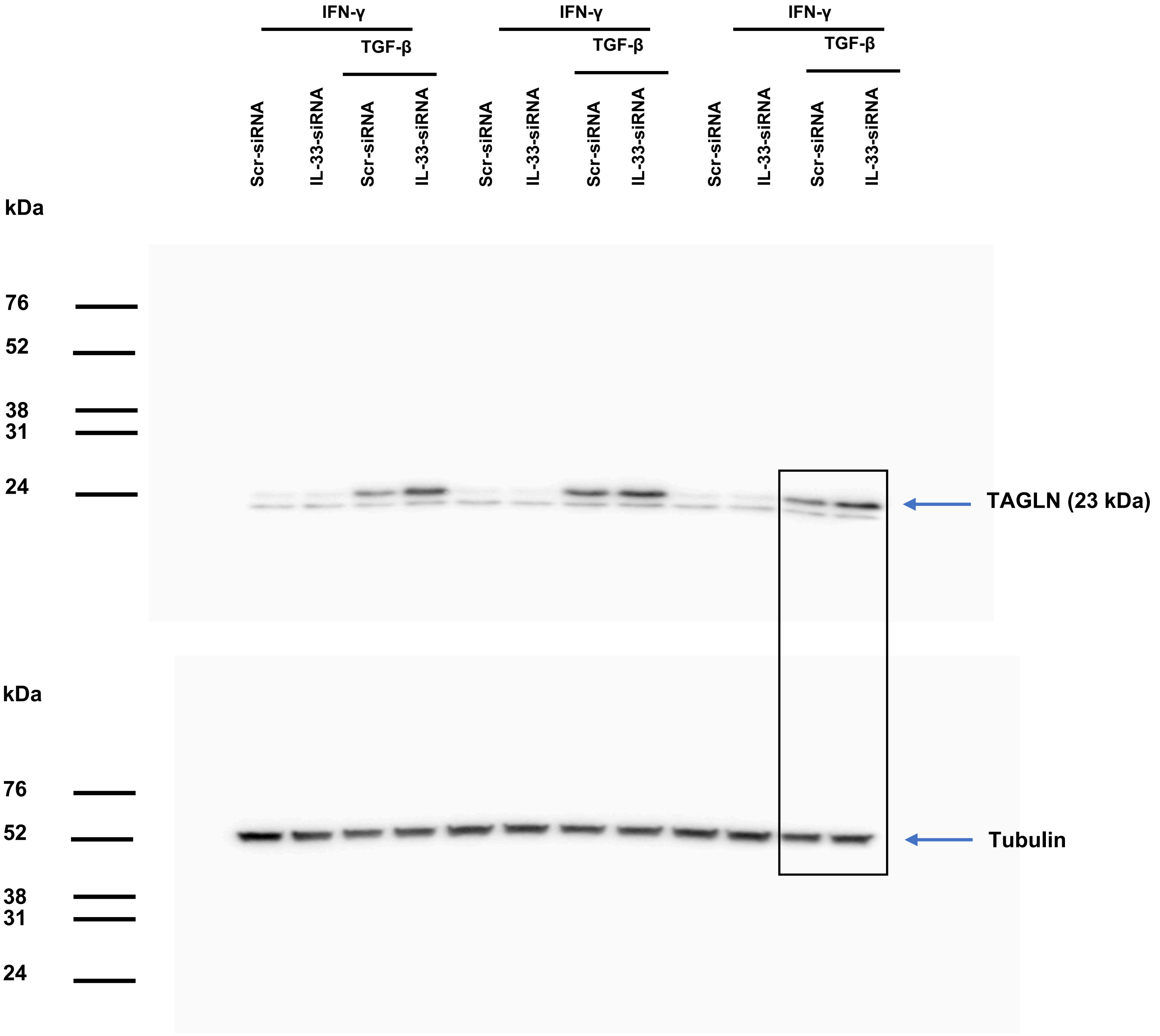

Gatti et al - Figure S7

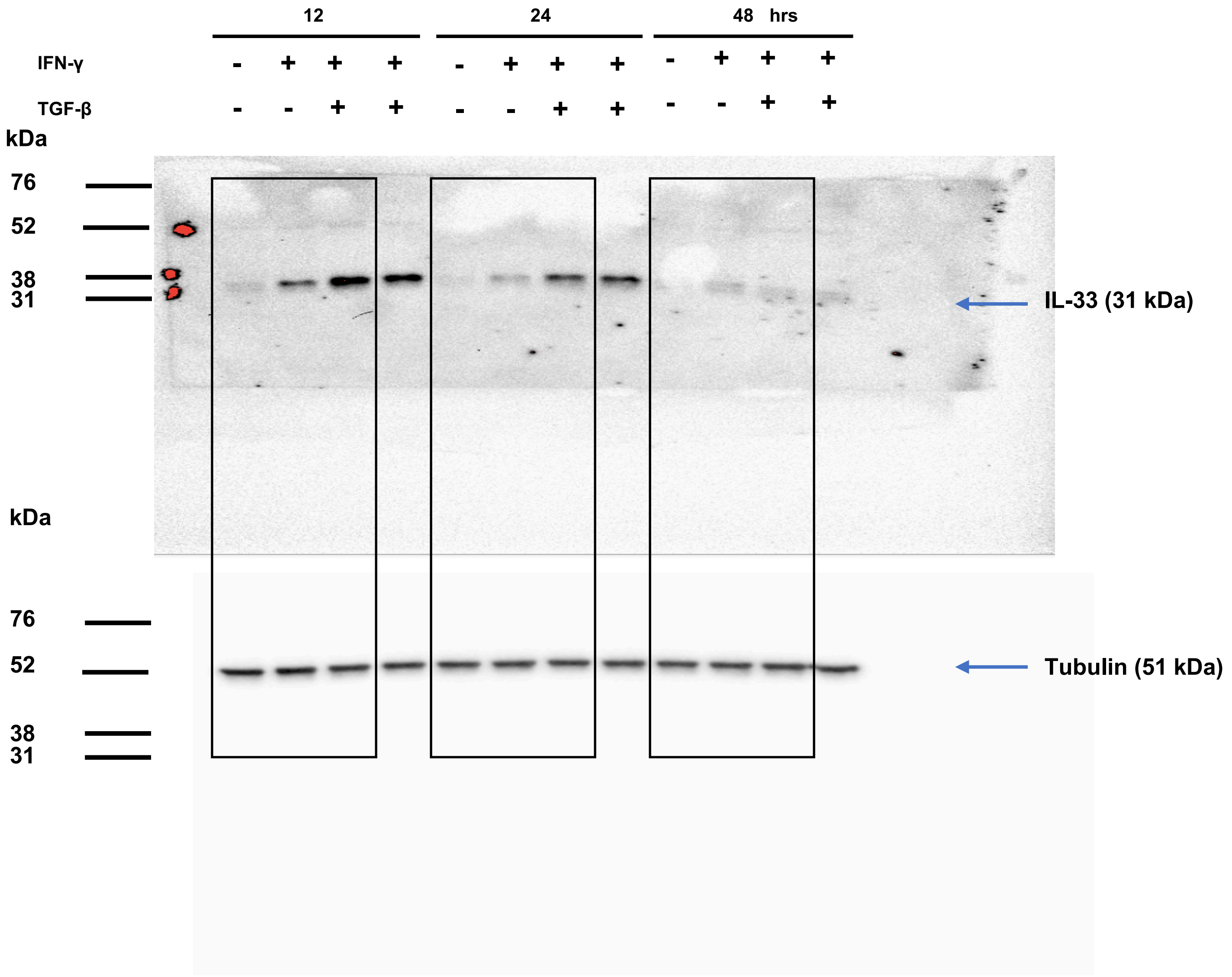

Table S1: Differentially regulated* probes in the contrast IL-33 KO vs WT on day 1 after unilateral ureteral obstruction

IlluminaID	SYMBOL	logFC	AveExpr	t	P.Value	fdr
ILMN_1259747	Il33	-2,36	8,15	- 14,98	0,0000	0,000
ILMN_2625893	Ces1d	-1,29	10,04	-5,91	0,0001	0,141
ILMN_2903540	Spp2	-1,14	9,85	-4,12	0,0015	0,190
ILMN_2443330	Ttr	-1,08	9,68	-4,26	0,0012	0,184
ILMN_2803249	Inmt	-1,03	8,80	-4,57	0,0007	0,155
ILMN_2734251	Dnase1	-1,02	10,63	-5,17	0,0002	0,145
ILMN_2873750	Gldc	-0,93	10,51	-4,35	0,0010	0,164
ILMN_2722579	Slc13a3	-0,87	10,08	-4,74	0,0005	0,145
ILMN_1238140	Ces1f	-0,86	10,93	-4,22	0,0012	0,185
ILMN_1218747	Gldc	-0,85	8,75	-3,80	0,0026	0,222
ILMN_2604310	Eci3	-0,83	9,78	-5,76	0,0001	0,141
ILMN_2706120	Ehhadh	-0,83	9,32	-3,72	0,0031	0,228
ILMN_2640116	Miox	-0,82	11,37	-3,63	0,0036	0,241
ILMN_2708491	Slc22a6	-0,80	11,85	-5,16	0,0003	0,145
ILMN_2870786	Upb1	-0,80	9,28	-4,60	0,0006	0,154
ILMN_2687507	Cyp2d9	-0,78	10,52	-3,88	0,0023	0,215
ILMN_2687014	Cyp2e1	-0,77	12,73	-4,90	0,0004	0,145
ILMN_2918312	Slc13a3	-0,76	9,46	-4,19	0,0013	0,185
ILMN_1216597	Slc25a25	-0,75	8,76	-4,81	0,0005	0,145
ILMN_2918732	Ugt3a1	-0,74	11,57	-4,29	0,0011	0,177
ILMN_2790496	Cyp4b1	-0,71	12,19	-6,29	0,0000	0,141
ILMN_2655795	Car4	-0,68	9,13	-3,85	0,0024	0,215
ILMN_2800986	Cyp2j11	-0,64	10,13	-3,61	0,0037	0,245
ILMN_2856537	Slc16a4	-0,63	8,01	-4,93	0,0004	0,145
ILMN_2785586	Fbp1	-0,62	11,31	-3,81	0,0026	0,219
ILMN_2863849	C1qtnf3	-0,62	7,89	-3,97	0,0019	0,204
ILMN_2725198	Azgp1	-0,61	8,47	-4,83	0,0004	0,145
ILMN_2699307	Hebp1	-0,61	9,19	-4,08	0,0016	0,194
ILMN_2837080	Azgp1	-0,60	10,47	-5,34	0,0002	0,145
ILMN_2777576	Cyp2j13	-0,59	8,58	-4,55	0,0007	0,155
ILMN_2870788	Upb1	-0,59	8,67	-3,63	0,0035	0,241
ILMN_2714031	Errfi1	-0,58	12,47	-4,91	0,0004	0,145
ILMN_3147331	Thnsl2	-0,57	9,37	-3,59	0,0039	0,246
ILMN_2675064	Adhfe1	-0,57	9,09	-5,06	0,0003	0,145
ILMN_2916782	Apom	-0,55	9,33	-4,53	0,0007	0,155
ILMN_2980663	Aqp1	-0,54	10,15	-3,59	0,0038	0,246
ILMN_3111685	Acsm3	-0,53	11,39	-3,74	0,0029	0,227
ILMN_1251401	Slc22a12	-0,53	10,40	-5,07	0,0003	0,145
ILMN_1213954	Sgk1	-0,52	10,90	-4,08	0,0016	0,194
ILMN_2493175	Tst	-0,52	9,71	-5,43	0,0002	0,145
ILMN_2769089	Serpinf2	-0,52	10,99	-3,57	0,0040	0,248

ILMN_2800732	Slc22a19	-0,51	9,13	-4,51	0,0008	0,156
ILMN_2885532	Cpt2	-0,51	10,22	-3,89	0,0022	0,214
ILMN_1256019	Acadm	-0,50	11,48	-5,08	0,0003	0,145
ILMN_2720751	Slc6a20b	-0,50	8,95	-3,59	0,0038	0,246
ILMN_1229964	Gstz1	-0,50	11,05	-3,72	0,0030	0,228
ILMN_1220761	Mpv17l	-0,50	9,63	-5,37	0,0002	0,145
ILMN_2657685	Aass	-0,49	8,27	-4,31	0,0011	0,174
ILMN_2592010	Fam195a	-0,49	9,24	-4,90	0,0004	0,145
ILMN_2688639	Pxmp4	-0,48	9,44	-4,38	0,0009	0,162
ILMN_2820305	Mep1b	-0,48	7,98	-3,65	0,0034	0,237
ILMN_3001827	Serpinf2	-0,48	9,68	-3,79	0,0027	0,224
ILMN_2692723	Lpl	-0,47	12,21	-4,11	0,0015	0,191
ILMN_2794258	Mpv17l	-0,47	9,49	-4,75	0,0005	0,145
ILMN_2619330	Plk3	-0,47	8,50	-3,81	0,0026	0,219
ILMN_2599348	Mfsd4b5	-0,46	10,47	-4,62	0,0006	0,154
ILMN_2677728	Abhd3	-0,46	8,66	-4,96	0,0004	0,145
ILMN_2896248	F13b	-0,46	9,46	-3,62	0,0036	0,242
ILMN_1241892	Sod3	-0,46	9,04	-5,82	0,0001	0,141
ILMN_2744603	Gchfr	-0,45	8,47	-4,43	0,0009	0,157
ILMN_2637180	C8g	-0,45	8,54	-4,08	0,0016	0,194
ILMN_2658054	Dhrs4	-0,44	9,11	-4,86	0,0004	0,145
ILMN_3152079	Slc22a18	-0,44	11,54	-3,68	0,0033	0,234
ILMN_2925947	Abat	-0,43	8,55	-3,70	0,0032	0,234
ILMN_1221787	Tcn2	-0,43	11,25	-4,92	0,0004	0,145
ILMN_2589651	Anpep	-0,43	7,94	-4,34	0,0010	0,166
ILMN_2591076	Slc22a19	-0,42	8,44	-3,88	0,0023	0,215
ILMN_2802487	Defb29	-0,41	9,96	-3,73	0,0030	0,227
ILMN_1248316	Ptgds	-0,41	7,51	-3,60	0,0038	0,246
ILMN_2740149	Chpt1	-0,41	11,89	-5,57	0,0001	0,141
ILMN_2634667	Mep1b	-0,41	7,38	-3,90	0,0022	0,213
ILMN_2609052	Dynll2	-0,40	7,86	-4,54	0,0007	0,155
ILMN_1256103	Kyat3	-0,39	8,74	-4,90	0,0004	0,145
ILMN_2645295	Fam132a	-0,39	8,89	-3,89	0,0023	0,214
ILMN_1234072	Pdlim1	0,38	9,08	3,98	0,0019	0,203
ILMN_2663249	Slamf9	0,39	8,86	3,89	0,0022	0,214
ILMN_1223552	Fbn1	0,39	8,59	5,52	0,0001	0,141
ILMN_3163159	Gng5	0,39	8,45	5,13	0,0003	0,145
ILMN_2755660	Ptma	0,40	10,06	5,53	0,0001	0,141
ILMN_2733330	Rps3a1	0,42	10,09	4,14	0,0014	0,190
ILMN_1226666	Cnn2	0,43	7,98	3,97	0,0019	0,204
ILMN_2887065	Mvp	0,43	11,20	3,84	0,0025	0,218
ILMN_1253806	Col1a2	0,43	7,79	4,05	0,0017	0,194
ILMN_3067068	Tmsb10	0,44	12,00	4,67	0,0006	0,152
ILMN_2591425	Gucy1a3	0,45	9,10	3,67	0,0033	0,234
ILMN_2793062	Rasl11b	0,45	9,20	3,90	0,0022	0,213
ILMN_1249727	Car13	0,45	9,07	4,93	0,0004	0,145

ILMN_2923445	Acta2	0,46	9,54	4,22	0,0013	0,185
ILMN_1242313	Spaca7	0,49	7,57	6,93	0,0000	0,118
ILMN_2759484	C3	0,49	12,25	4,14	0,0014	0,190
ILMN_1259215	Serpina10	0,51	11,54	4,22	0,0013	0,185
ILMN_2899041	Mmp3	0,51	8,12	4,42	0,0009	0,157
ILMN_2710353	Acta2	0,52	9,34	5,69	0,0001	0,141
ILMN_1258629	Col3a1	0,54	8,37	4,75	0,0005	0,145
ILMN_2710354	Acta2	0,58	9,55	7,21	0,0000	0,117
ILMN_2693895	Acta2	0,59	12,24	7,15	0,0000	0,117
ILMN_2687872	Col1a1	0,61	9,03	5,28	0,0002	0,145
ILMN_2778655	Vcam1	0,68	9,03	4,34	0,0010	0,165
ILMN_1248830	Hist1h2an	0,74	8,20	3,72	0,0030	0,228
ILMN_2601519	Aoc1	0,89	10,60	5,40	0,0002	0,145

** Threshold fdr<0.25, fold change>1.3*
The probes detect 89 unique genes, including Il33.

Table S2: Gene Ontology analysis of Differentially regulated genes* in the contrast IL-33 KO vs WT on day 1 after unilateral ureteral obstruction

**Positive enrichment in IL33 KO kidney**				
**Annotation Cluster 1, Enrichment Score 3.7**		**count**	**p-value**	**Benjamini**
GOTERM_MF_DIRECT	extracellular matrix structural constituent	4	1.3E-5	8.8E-4
UP_KEYWORDS	Extracellular matrix	5	7.1E-5	5.4E-3
GOTERM_BP_DIRECT	skeletal system development	4	2.5E-4	4.6E-2
GOTERM_CC_DIRECT	extracellular matrix	5	2.9E-4	6.1E-3
GOTERM_CC_DIRECT	proteinaceous extracellular matrix	5	3.8E-4	6.0E-3
GOTERM_BP_DIRECT	cellular response to amino acid stimulus	4	5.1E-4	4.7E-2
UP_KEYWORDS	Calcium	6	1.0E-3	2.5E-2
**Annotation Cluster 2, Enrichment score 3.02**		**count**	**p-value**	**Benjamini**
UP_SEQ_FEATURE	propeptide:N-terminal propeptide	3	2.1E-5	3.6E-3
UP_SEQ_FEATURE	propeptide:C-terminal propeptide	3	3.0E-5	2.5E-3
INTERPRO	Fibrillar collagen, C-terminal	3	5.4E-5	4.8E-3
UP_SEQ_FEATURE	domain:Fibrillar collagen NC1	3	6.4E-5	3.6E-3
GOTERM_MF_DIRECT	platelet-derived growth factor binding	3	8.2E-5	2.7E-3
SMART	COLFI	3	1.4E-4	3.5E-3
GOTERM_BP_DIRECT	collagen fibril organization	3	9.3E-4	5.7E-2
INTERPRO	Collagen triple helix repeat	3	2.7E-3	1.1E-1
GOTERM_BP_DIRECT	blood vessel development	3	3.0E-3	1.1E-1
UP_KEYWORDS	Collagen	3	3.1E-3	4.5E-2
UP_KEYWORDS	Hydroxylation	3	3.3E-3	4.1E-2
GOTERM_CC_DIRECT	collagen trimer	3	3.8E-3	4.0E-2
KEGG_PATHWAY	Protein digestion and absorption	3	9.2E-3	2.2E-1
KEGG_PATHWAY	ECM-receptor interaction	3	9.2E-3	2.2E-1
KEGG_PATHWAY	Amoebiasis	3	1.6E-2	2.5E-1
KEGG_PATHWAY	Focal adhesion	3	4.6E-2	4.0E-1
**Annotation Cluster 3, Enrichment score 2.35**		**count**	**p-value**	**Benjamini**
UP_KEYWORDS	Glycoprotein	11	1.6E-3	3.0E-2
UP_SEQ_FEATURE	signal peptide	11	2.1E-3	8.3E-2
UP_KEYWORDS	Signal	11	6.2E-3	6.5E-2
UP_SEQ_FEATURE	glycosylation site:N-linked (GlcNAc...)	10	1.9E-2	4.7E-1

Negative enrichment in IL33 KO kidney				
**Annotation Cluster 4, Enrichment score 5.08**		**count**	**p-value**	**Benjamini**
UP_KEYWORDS	Oxidoreductase	13	1.9E-7	2.6E-5
GOTERM_MF_DIRECT	oxidoreductase activity	11	3.6E-5	3.7E-3
GOTERM_BP_DIRECT	oxidation-reduction process	11	8.0E-5	2.9E-2
**Annotation Cluster 5, Enrichment score 3.29**		**count**	**p-value**	**Benjamini**
UP_KEYWORDS	Secreted	16	5.4E-5	2.4E-3
GOTERM_CC_DIRECT	extracellular region	16	3.4E-4	7.6E-3
UP_SEQ_FEATURE	signal peptide	19	7.6E-3	3.4E-1
**Annotation Cluster 6, Enrichment score 2.97**		**count**	**p-value**	**Benjamini**
UP_KEYWORDS	Peroxisome	5	2.4E-4	8.0E-3
GOTERM_CC_DIRECT	peroxisome	5	8.3E-4	1.5E-2
KEGG_PATHWAY	Peroxisome	4	6.1E-3	9.6E-2
**Annotation Cluster 7, Enrichment score 2.84**		**count**	**p-value**	**Benjamini**
INTERPRO	Cytochrome P450, E-class, group I	5	1.2E-4	9.6E-3
INTERPRO	Cytochrome P450, conserved site	5	2.1E-4	1.1E-2
INTERPRO	Cytochrome P450	5	2.9E-4	1.1E-2
GOTERM_MF_DIRECT	heme binding	6	3.4E-4	2.2E-2
GOTERM_MF_DIRECT	oxidoreductase activity, acting on paired donors, with incorporation or reduction of molecular oxygen, reduced flavin or flavoprotein as one donor, and incorporation of one atom of oxygen	4	4.0E-4	2.0E-2
UP_KEYWORDS	Monooxygenase	5	5.5E-4	1.2E-2
GOTERM_MF_DIRECT	arachidonic acid epoxygenase activity	4	5.6E-4	2.3E-2
GOTERM_MF_DIRECT	iron ion binding	6	8.0E-4	2.3E-2
GOTERM_MF_DIRECT	steroid hydroxylase activity	4	8.5E-4	2.1E-2
UP_KEYWORDS	Heme	5	1.4E-3	1.7E-2
GOTERM_MF_DIRECT	oxidoreductase activity, acting on paired donors, with incorporation or reduction of molecular oxygen	4	4.8E-3	6.3E-2

GOTERM_BP_DIRECT	arachidonic acid metabolic process	3	6.6E-3	2.9E-1
COG_ONTOLOGY	Secondary metabolites biosynthesis, transport, and catabolism	5	1.2E-2	3.5E-2
KEGG_PATHWAY	Linoleic acid metabolism	3	2.1E-2	1.2E-1
KEGG_PATHWAY	Serotonergic synapse	3	1.2E-1	4.3E-1
**Annotation Cluster 8, Enrichment score 2.62**		**count**	**p-value**	**Benjamini**
UP_KEYWORDS	Pyridoxal phosphate	4	6.5E-4	1.3E-2
GOTERM_MF_DIRECT	pyridoxal phosphate binding	4	8.9E-4	2.0E-2
INTERPRO	Pyridoxal phosphate-dependent transferase	3	7.7E-3	1.6E-1
INTERPRO	Pyridoxal phosphate-dependent transferase, major region, subdomain 1	3	7.7E-3	1.6E-1
**Annotation Cluster 9, Enrichment score 2.41**		**count**	**p-value**	**Benjamini**
GOTERM_MF_DIRECT	inorganic anion exchanger activity	3	2.3E-3	4.2E-2
GOTERM_MF_DIRECT	sodium-independent organic anion transmembrane transporter activity	3	3.9E-3	5.5E-2
GOTERM_BP_DIRECT	sodium-independent organic anion transport	3	4.0E-3	2.6E-1
INTERPRO	General substrate transporter	3	6.3E-3	1.5E-1
**Annotation Cluster 10, Enrichment score 2.38**		**count**	**p-value**	**Benjamini**
KEGG_PATHWAY	beta-Alanine metabolism	4	4.2E-4	1.4E-2
KEGG_PATHWAY	Propanoate metabolism	3	6.5E-3	8.3E-2
KEGG_PATHWAY	Valine, leucine and isoleucine degradation	3	2.6E-2	1.2E-1
**Annotation Cluster 11, Enrichment score 1.87**		**count**	**p-value**	**Benjamini**
KEGG_PATHWAY	PPAR signaling pathway	4	5.5E-3	1.1E-1
KEGG_PATHWAY	Fatty acid degradation	3	2.1E-2	1.3E-1
KEGG_PATHWAY	Fatty acid metabolism	3	2.2E-2	1.2E-1
**Annotation Cluster 12, Enrichment score 1.54**		**count**	**p-value**	**Benjamini**
GOTERM_MF_DIRECT	oxidoreductase activity, acting on paired	4	4.8E-3	6.3E-2

	donors, with incorporation or reduction of molecular oxygen			
UP_SEQ_FEATURE	metal ion-binding site:Iron (heme axial ligand)	3	5.2E-2	8.3E-1
GOTERM_MF_DIRECT	monooxygenase activity	3	5.3E-2	4.4E-1
UP_KEYWORDS	Microsome	3	5.4E-2	2.6E-1

** Differentially regulated probes are listed in table S1.*

Table S3: Differentially regulated* probes in human fibroblasts treated with siRNA targeting IL-33 compared to fibroblasts treated with scrambled siRNA

IlluminaID	SYMBOL	IFNγ alone		IFNγ and TGFβ	
		logFC	adj.P.Val	logFC	adj.P.Val
ILMN_1809099	IL33	-1,32	1,06E-06	-2,03	2,66E-09
ILMN_1713934	LITAF	-1,72	2,41E-09	-1,69	2,66E-09
ILMN_2279961	LAMP2	-1,62	1,20E-09	-1,52	2,66E-09
ILMN_1795991	RTCB	-1,15	1,20E-09	-1,25	7,16E-10
ILMN_2212909	MELK	-1,26	2,14E-07	-1,22	2,74E-07
ILMN_1771593	RRM1	-0,94	4,85E-06	-1,00	2,28E-06
ILMN_1807833	HM13	-0,94	2,70E-07	-0,94	2,71E-07
ILMN_2149164	SFRP1	-0,43	0,0915	-0,91	0,0002
ILMN_1768662	UCK2	-0,64	0,0017	-0,84	0,0001
ILMN_1813475	HERC2	-0,79	1,13E-07	-0,82	6,75E-08
ILMN_1675085	UBA6	-0,80	2,16E-06	-0,80	2,07E-06
ILMN_1737857	GTF2B	-0,74	4,64E-06	-0,79	1,98E-06
ILMN_2160929	FEN1	-0,77	9,26E-06	-0,79	6,60E-06
ILMN_1772964	CCL8	-0,78	0,0013	-0,78	0,0012
ILMN_1665538	SKP2	-0,86	7,02E-08	-0,78	2,08E-07
ILMN_1800225	PPARG	-0,68	1,90E-05	-0,75	5,76E-06
ILMN_2111187	ELOVL6	-1,08	2,05E-05	-0,75	0,0009
ILMN_1725485	RGS17	-0,69	0,0227	-0,69	0,0223
ILMN_2126055	ASB5	-0,52	0,0269	-0,68	0,0036
ILMN_2182198	ICT1	-0,67	8,39E-06	-0,68	5,78E-06
ILMN_2126038	STMN2	-0,59	0,0138	-0,68	0,0041
ILMN_1683456	CCL7	-0,67	0,0001	-0,67	0,0001
ILMN_1799516	DNAJC9	-0,65	2,16E-05	-0,65	1,95E-05
ILMN_1680955	AURKA	-0,64	0,0004	-0,64	0,0005
ILMN_1757387	UCHL1	-0,52	0,0151	-0,61	0,0039
ILMN_1687978	PHLDA1	-0,14	0,9254	-0,61	0,0493
ILMN_1756220	DDX18	-0,61	4,62E-06	-0,59	5,76E-06
ILMN_1690099	ITGB1BP1	-0,54	0,0016	-0,59	0,0006
ILMN_1738491	SNX30	-0,49	0,0023	-0,59	0,0004
ILMN_1784661	TMEM2	-0,67	5,21E-05	-0,55	0,0004
ILMN_1770338	TM4SF1	-0,71	0,0111	-0,54	0,0713
ILMN_1712888	HSPH1	-0,68	0,0177	-0,50	0,1053
ILMN_1782057	ATP8B2	-0,74	5,54E-05	-0,49	0,0031
ILMN_1666444	RBMS1	-0,63	5,11E-05	-0,49	0,0007
ILMN_1691570	METTL5	-0,61	8,15E-05	-0,40	0,0053
ILMN_2082209	TOX2	-0,60	0,0003	-0,35	0,0294
ILMN_1668535	JOSD1	-0,73	2,37E-05	-0,33	0,0376
ILMN_1744381	SERPINE1	-0,61	0,0129	-0,22	0,5866
ILMN_1771026	GARS	-0,61	0,0036	-0,21	0,5143
ILMN_1699695	TNFRSF21	0,61	0,0008	0,24	0,2909
ILMN_1736670	PPP1R3C	0,82	1,56E-05	0,24	0,2363
ILMN_1730794	SERTAD4	0,58	0,0007	0,26	0,1910

ILMN_3241665	SERTAD4-AS1	0,70	0,0033	0,34	0,2567
ILMN_2229877	PCDH18	0,61	0,0243	0,37	0,2675
ILMN_1656111	MYLIP	0,65	0,0003	0,41	0,0174
ILMN_1730940	KLHDC3	0,61	0,0002	0,50	0,0014
ILMN_1785660	SRPRA	0,60	0,0012	0,53	0,0037
ILMN_1729288	C1QTNF6	0,67	4,85E-06	0,55	4,19E-05
ILMN_1793287	DEXI	0,63	0,0006	0,58	0,0011
ILMN_2399896	SEC31A	0,50	0,0002	0,60	3,14E-05
ILMN_1668507	DDAH1	0,56	0,0002	0,62	7,11E-05
ILMN_1664861	ID1	0,05	0,9506	0,63	1,27E-05
ILMN_1674236	HSPB1	0,64	2,37E-05	0,63	3,14E-05
ILMN_1704154	TNFRSF19	0,71	4,32E-05	0,63	0,0001
ILMN_1751097	CREB3L2	0,51	0,0021	0,66	0,0001
ILMN_1813314	HIST1H2BK	0,76	0,0055	0,68	0,0136
ILMN_1727458	HDAC1	0,71	4,85E-06	0,69	6,48E-06
ILMN_1773079	COL3A1	0,67	0,0177	0,69	0,0153
ILMN_1679640	FXR1	0,67	1,86E-06	0,69	1,17E-06
ILMN_1768940	COL15A1	0,80	0,0360	0,70	0,0802
ILMN_1701461	TIMP3	0,80	0,0163	0,72	0,0326
ILMN_1800317	WNT5A	0,64	0,0250	0,76	0,0070
ILMN_1708006	MICB	0,82	2,68E-07	0,85	1,63E-07
ILMN_1774547	MPRIP	1,14	1,20E-09	1,19	1,14E-09

** Threshold adjusted p-value<0,05, fold change>1,5*

Table S4: Gene Ontology analysis of Differentially regulated genes* in the contrast siIL33-treated vs SCR-treated fibroblasts

**Positive enrichment in siIL33-treated fibroblasts (25 genes)**				
**Annotation Cluster 1, Enrichment Score 1.99**		**count**	**p-value**	**Benjamini**
INTERPRO	Collagen triple helix repeat	3	4.2E-3	2.7E-1
GOTERM_CC_DIRECT	extracellular space	7	4.3E-3	2.3E-1
GOTERM_CC_DIRECT	collagen trimer	3	5.5E-3	1.1E-1
UP_KEYWORDS	Collagen	3	5.8E-3	2.3E-1
UP_KEYWORDS	Secreted	5	1.9E-1	8.8E-1
**Annotation Cluster 2, Enrichment score 1.31**		**count**	**p-value**	**Benjamini**
UP_KEYWORDS	Extracellular matrix	4	3.2E-3	2.5E-1
GOTERM_CC_DIRECT	extracellular space	7	4.3E-3	2.3E-1
GOTERM_CC_DIRECT	extracellular matrix	4	5.2E-3	1.5E-1
GOTERM_CC_DIRECT	endoplasmic reticulum lumen	3	2.2E-2	2.9E-1
UP_SEQ_FEATURE	signal peptide	9	3.5E-2	9.9E-1
GOTERM_CC_DIRECT	proteinaceous extracellular matrix	3	4.1E-2	4.1E-1
UP_KEYWORDS	Secreted	5	1.9E-1	8.8E-1
UP_KEYWORDS	Disulfide bond	7	2.0E-1	8.7E-1
GOTERM_CC_DIRECT	extracellular exosome	6	2.4E-1	8.9E-1
GOTERM_CC_DIRECT	extracellular region	4	3.1E-1	8.5E-1
UP_KEYWORDS	Disease mutation	4	5.9E-1	9.9E-1

**Negative enrichment in siIL33-treated fibroblasts (38 genes)**				
**Annotation Cluster 3, Enrichment score 2.82**		**count**	**p-value**	**Benjamini**
UP_KEYWORDS	ATP binding	10	5.1E-4	5.9E-2
GOTERM_MF_DIRECT	ATP binding	10	2.2E-3	2.2E-1
UP_KEYWORDS	Nucleotide-binding	10	3.0E-3	1.6E-1
**Annotation Cluster 4, Enrichment score 1.69**		**count**	**p-value**	**Benjamini**
GOTERM_BP_DIRECT	cellular response to interleukin-1	3	9.6E-3	8.5E-1
GOTERM_BP_DIRECT	cellular response to tumor necrosis factor	3	2.2E-2	8.8E-1
GOTERM_MF_DIRECT	heparin binding	3	4.1E-2	7.0E-1

** Differentially regulated genes are listed in heatmap in Figure 5F.*

Table S5: Gene Set Enrichment Analysis report for the contrast siIL33-treated vs SCR-treated fibroblasts, using fibroblast phenotype gene sets

Name	Size	ES	NES	NOM p-val	FDR q-val	FWER p-val	Rank at max	Leading edge
ICAF vs Quiescent (TNF/IFN)	443	-0,40	-1.40	<0.001	0.043	0.043	2455	tags=16%, list=7%, signal=17%
ICAF vs Quiescent (IFN)	443	-0,36	-1.32	<0.001	0.087	0.119	2468	tags=15%, list=7%, signal=16%
MFBL vs ICAF (IFN)	427	0,33	1.28	<0.001	0.162	0.145	3912	tags=16%, list=11%, signal=18%

Threshold p<0,05 and false discovery rate 25%
